# Blood‐derived microRNAs are related to cognitive domains in the general population

**DOI:** 10.1002/alz.14197

**Published:** 2024-08-29

**Authors:** Konstantinos Melas, Valentina Talevi, Mohammed Aslam Imtiaz, Rika Etteldorf, Santiago Estrada, Dennis M. Krüger, Tonatiuh Pena‐Centeno, N. Ahmad Aziz, André Fischer, Monique M. B. Breteler

**Affiliations:** ^1^ Population Health Sciences German Centre for Neurodegenerative Diseases (DZNE) Bonn Germany; ^2^ AI in Medical Imaging German Centre for Neurodegenerative Diseases (DZNE) Bonn Germany; ^3^ Department for Epigenetics and Systems Medicine in Neurodegenerative Diseases German Center for Neurodegenerative Diseases (DZNE) Göttingen Germany; ^4^ Bioinformatics Unit German Centre for Neurodegenerative Diseases (DZNE) Göttingen Germany; ^5^ Department of Neurology Faculty of Medicine University of Bonn Bonn Germany; ^6^ Department for Psychiatry and Psychotherapy University Medical Center Göttingen Göttingen Germany; ^7^ Cluster of Excellence MBExC University of Göttingen & University Medical Center Goettingen Göttingen Germany; ^8^ Institute for Medical Biometry Informatics and Epidemiology (IMBIE) Faculty of Medicine University of Bonn Bonn Germany

**Keywords:** biomarker, cognition, cortical thickness, microRNA, miR‐10401‐3p, miR‐125b‐5p, miR‐128‐3p, miR‐134‐5p, miR‐192‐5p, miR‐215‐5p, miR‐4677‐5p, miR‐4732‐3p, miR‐92a‐3p, miR‐92b‐3p, population‐based

## Abstract

**INTRODUCTION:**

Blood‐derived microRNAs (miRNAs) are potential candidates for detecting and preventing subclinical cognitive dysfunction. However, replication of previous findings and identification of novel miRNAs associated with cognitive domains, including their relation to brain structure and the pathways they regulate, are still lacking.

**METHODS:**

We examined blood‐derived miRNAs and miRNA co‐expression clusters in relation to cognitive domains, structural magnetic resonance imaging measures, target gene expression, and genetic variants in 2869 participants of a population‐based cohort.

**RESULTS:**

Five previously identified and 14 novel miRNAs were associated with cognitive domains. Eleven of these were also associated with cortical thickness and two with hippocampal volume. Multi‐omics analysis showed that certain identified miRNAs were genetically influenced and regulated genes in pathways like neurogenesis and synapse assembly.

**DISCUSSION:**

We identified miRNAs associated with cognitive domains, brain regions, and neuronal processes affected by aging and neurodegeneration, making them promising candidate blood‐based biomarkers or therapeutic targets of subclinical cognitive dysfunction.

**Highlights:**

- We investigated the association of blood‐derived microRNAs with cognitive domains.
- Five previously identified and 14 novel microRNAs were associated with cognition.
- Eleven cognition‐related microRNAs were also associated with cortical thickness.
- Identified microRNAs were linked to genes associated with neuronal functions.
- Results provide putative biomarkers or therapeutic targets of cognitive aging.

## 1 BACKGROUND

Mitigating the impact of cognitive aging remains one of the biggest challenges of the twenty‐first century.^1^ Early detection of subclinical cognitive dysfunction is necessary to enable interventions at the earliest stages of its pathophysiology.^2^ Currently, tailored neuropsychological test batteries are the gold standard for detecting cognitive dysfunction. However, such batteries can be time consuming, require experienced examiners, and are dependent on individual traits such as language and cultural background. Moreover, their sensitivity in the very early stages of cognitive dysfunction, and in highly educated persons, is limited.^3^ The identification of blood‐based molecular biomarkers of subclinical cognitive dysfunction could facilitate earlier and more reliable detection of individuals at risk of accelerated cognitive aging or neurodegeneration.^4^ Additionally, such biomarkers might point to specific pathophysiologic processes involved in cognitive aging, and thus help the development of targeted interventions.

Various blood‐based biomarkers have been investigated for the early detection of cognitive dysfunction, including neurodegenerative,^4^ lipidomic, inflammatory,^5^ and proteomic^6^ markers. A highly promising line of research focuses on microRNAs (miRNAs).^7^, ^8^ These small, endogenous, non‐coding RNAs bind to target messenger RNAs, mainly through base pairing, leading to degradation of these messenger RNAs or inhibition of their translation. A single miRNA can have several physiological effects through the regulation of multiple genes.^9^ Additionally, miRNAs are highly expressed in the brain, where they regulate functions like synaptic plasticity and memory. Brain‐enriched miRNAs can cross the blood‐brain barrier and enter the peripheral circulation, where they remain stable for extended periods.^8^ Thus, miRNAs have been investigated for the diagnosis and treatment of neurodegenerative diseases.^10^ For example, circulating miRNA‐based panels were shown to have high accuracy in diagnosing Alzheimer's disease (AD), and one such diagnostic panel has entered phase I of clinical development.^11^ Conversely, miRNA‐based therapeutics have been hindered by the incomplete characterization of the functions of tested miRNAs, which can lead to unforeseen side effects.^11^

To assess whether blood‐derived miRNAs can be used for early detection or prevention of cognitive dysfunction, their association with cognition must be investigated in non‐demented individuals, and their functions must be better understood. In the past, a few population‐based studies identified plasma or whole‐blood miRNA signatures associated with general cognitive function and decline,^12^, ^13^ verbal memory changes longitudinally,^14^ or multiple cognitive domains cross‐sectionally.^15^ A recent study identified a three‐miRNA signature related to cognitive function and decline, by combining population‐level and animal model data.^16^ However, most of these studies had relatively small samples, potentially limiting their capability to detect miRNAs with weaker effect sizes. Specifically, the largest study included 1615 participants,^12^ while the rest had sample sizes ranging from 132^16^ to 830^15^ participants. Moreover, the only study that examined more than one cognitive domain evaluated only a small number (*n* = 38) of miRNAs previously linked to AD.^15^ Thus, there is a need for studies combining larger sample sizes, multiple cognitive domains, and the untargeted evaluation of many miRNA transcripts. In addition, it is worth noting that only five miRNAs (miR‐4732‐3p, miR‐363‐3p, miR‐181a‐5p, miR‐146a‐5p, and let‐7c‐5p) overlapped among at least two of these studies. This reflects a greater problem in miRNA research. The large heterogeneity in miRNA sampling and quantification methods often leads to inconsistent results among studies, which highlights the need for replication.^17^ Last, none of these studies examined the association of cognition‐related miRNAs with brain structure or gene expression. This could provide information on key miRNA‐regulated biological pathways, enabling their use as therapeutic targets against cognitive decline.

Here, we aimed to first identify blood‐circulating miRNAs associated with the major domains of cognitive function, by replicating the results of previous studies and identifying novel associations in a population‐based cohort consisting of 2869 individuals. Next, we determined which of the identified cognition‐related miRNAs were associated with brain structure. Leveraging individual‐level gene expression data in a functional genomics approach, we elucidated the molecular pathways through which the identified miRNAs could affect cognitive function and brain structure. Last, we detected genetic variants influencing the expression of identified miRNAs and examined potential causal associations between miRNAs and cognition in a Mendelian randomization framework.

## 2 METHODS

### 2.1 Study design

Our analysis was based on cross‐sectional baseline data from the Rhineland Study, an ongoing prospective, population‐based cohort study in Bonn, Germany. All residents of two geographically defined areas in Bonn, aged ≥ 30 years, were invited to participate in the study. The only exclusion criterion was an insufficient command of the German language to provide informed consent. One of the main goals of the Rhineland Study is to investigate biomarkers and determinants of aging and age‐related diseases in the general population, following a deep‐phenotyping approach. Each participant undergoes a comprehensive 7‐hour examination protocol, which includes detailed questionnaires, brain imaging, neuropsychological examinations, and blood sample analyses.

### 2.2 Standard protocol approvals, registrations, and participant consent

Approval to undertake the study was obtained from the ethics committee of the University of Bonn, Medical Faculty. The study is carried out in accordance with the recommendations of the International Conference on Harmonization Good Clinical Practice standards. We obtained written informed consent from all participants in accordance with the Declaration of Helsinki.

### 2.3 Study population

Biomaterial was selected from the first 3000 participants of the Rhineland Study who provided blood samples for miRNA sequencing. MiRNA expression data were missing due to technical issues (*n* = 33) or exclusion during quality control (*n* = 38). From the remaining 2929, we excluded 60 participants because of missing cognition data (due to: contraindications/exclusion criteria, *n* = 7; participant refusal, *n* = 3; technical issues, *n* = 3; exclusion during quality control, *n* = 9; a combination of the above, *n* = 38), leaving 2869 participants with both miRNA expression and cognitive performance data (“cognition dataset”). Brain imaging data were available for a subset of 2045 participants of the cognition dataset (brain imaging data missing due to: contraindications/exclusion criteria, *n* = 452; participant refusal, *n* = 330; other/unknown reasons, *n* = 42). Gene expression and blood cell count data were available for a subset of 2138 participants of the cognition dataset (gene expression data missing due to: technical issues, *n* = 475; exclusion during quality control, *n* = 129; blood cell count data missing due to: technical issues, *n* = 126; unknown reasons, *n* = 1). The overlap of the subset with complete MRI data and the subset with complete gene expression and blood cell count data consisted of 1534 participants.

**RESEARCH IN CONTEXT**

1. **Systematic review**: We searched PubMed for studies linking circulating microRNAs to cognition, mild cognitive impairment or Alzheimer's disease. Previous studies had smaller samples and mostly focused on aggregate measures of cognitive performance. Because of heterogeneity in data collection and analysis methods, and increasing number of identified microRNAs, there is a need for replication and extension of previous findings.
2. **Interpretation**: In a population‐based study of 2869 participants we partially replicated previously suggested microRNAs, and identified novel ones, associated with specific cognitive domains. Magnetic resonance imaging and functional genomics analyses showed that identified microRNAs were related to brain regions and biological pathways important for cognitive processes, suggesting that these microRNAs are causally related to cognition.
3. **Future directions**: Further validation of our findings in other cohorts, longitudinal studies, and diverse populations can help assess the usefulness of identified microRNAs as biomarkers for cognition. Moreover, experimental studies should investigate identified microRNAs as therapeutic targets against cognitive aging.

### 2.4 Blood sample acquisition and RNA isolation

Blood samples were collected between 7:00 am and 9:45 am from an antecubital or dorsal hand vein. On some occasions, blood sample collection was performed on a different visit than the cognitive evaluation. The time difference between the two visits was usually < 1 month. Whole blood samples for miRNA and gene (messenger RNA) expression sequencing were collected in PAXgene Blood RNA tubes (PreAnalytix/Qiagen). These tubes contain a stabilizing solution, which causes immediate lysis of all cells and prevents changes to RNA expression profiles. The PAXgene tubes were stored at −80°C and were thawed and incubated at room temperature to increase RNA yield before sequencing. Total RNA was isolated according to the manufacturer's instructions using the PAXgene Blood miRNA Kit and following the automated purification protocol (PreAnalytix/Qiagen).

### 2.5 MiRNA and gene expression measurement

We performed sequencing of miRNAs on the Illumina HiSeq 2000 over 44 batches (plates), and RNA sequencing on the NovaSeq6000 platform over 28 batches (plates). Detailed information on our miRNA and gene sequencing and analysis pipeline is provided in Methods S1 in supporting information. MiRNAs and genes with overall mean expression levels > 15 reads and expressed in at least 5% of the participants were included for further analysis, resulting in a total of 415 miRNAs and 11,325 genes. These 415 miRNAs were derived from 298 unique miRNA precursors located across the genome. Raw counts were normalized and transformed using the varianceStabilizingTransformation function from DESeq2 (v1.30.1), which includes a log2 transformation.^18^ Mean normalized counts of sequenced miRNAs ranged from 3.09 (for miR‐4707‐3p) to 21.34 (for miR‐486‐5p). As a last step, miRNA and gene expression data were adjusted for sequencing batch effects by creating a linear regression model, with miRNA normalized counts as the dependent variable and sequencing plate number as the independent variable, and extracting the model residuals.

### 2.6 Genotyping

Genotyping was performed on the Omni 2.5 Exome Array, using GenomeStudio (version 2.0.5) for genotype calling. We used PLINK (version 1.9) to exclude single nucleotide polymorphisms (SNPs) based on poor genotyping rate (< 99%), Hardy–Weinberg disequilibrium (*p* < 1e−6), poor sample call rate (< 95%), abnormal heterozygosity, cryptic relatedness, and sex mismatch. Population substructure was analyzed through EIGENSOFT v7.2.1.0.^19^ To account for systematic variations in allele frequencies due to different ethnic backgrounds, we excluded participants of non‐European descent based on the genetic principal component (*n* = 236). We imputed genotypes using IMPUTE v2,^20^ with 1000 Genomes phase 3 v5 as the reference panel.^21^

### 2.7 Assessment of cognitive function

The neuropsychological test battery of the Rhineland Study has been described in detail elsewhere.^22^ Examined domains included working memory, episodic verbal memory, processing speed, executive function, and crystallized intelligence. Working memory was assessed with the orally performed Digit Span forward and backward task, and the touchpad‐based Corsi block‐tapping test, based on the Psychology Experiment Building Language battery.^23^ Episodic verbal memory was evaluated with the Auditory Verbal Learning and Memory test with a list length of 15 words.^24^ Assessment of processing speed was based on a numbers‐only Trail‐Making Test (Trail‐Making Test, Part A), and prosaccade latency (time needed to initiate a saccade), derived from an eye movement test battery.^25^ We examined executive function using the antisaccade error rate (percentage of trials in which the participant made a direction error) from the same battery, combined with a categorical word fluency task (animals), and a number‐and‐letters Trail‐Making Test (Trail‐Making Test, Part B). Last, we measured crystallized intelligence with the 37‐item Mehrfachwahl‐Wortschatz‐Intelligenztest.^26^ Cognitive domain performance scores were created by averaging the *z* scores of the tests contributing to each domain. The creation of these *z* scores was based on data from the first 5000 participants of the Rhineland Study. Additionally, we averaged the *z* scores for working memory, episodic verbal memory, processing speed, and executive function to create a global cognition composite score. All examinations were administered in German language, following a standardized procedure by certified study technicians.

### 2.8 Image acquisition and brain segmentation

Participants underwent a 1‐hour examination protocol in 3T magnetic resonance imaging (MRI) scanners (Siemens Prisma Magnetom) equipped with a 64‐channel head‐neck coil.^27^ During the MRI section, a T1‐weighted multi‐echo magnetization‐prepared rapid gradient echo (MPRAGE) image was acquired.^28^ T1‐weighted images were processed with the standard FreeSurfer (version 6.0) recon‐all pipeline^29^ to obtain the brain imaging phenotypes. For our analysis of MRI data, we evaluated regions relevant to cognitive aging and neurodegeneration, namely mean and regional cortical thickness, and hippocampal volume.^30^ In addition, we evaluated total brain volume as a general indicator of brain health. Regional cortical thickness was evaluated for the 31 cortical regions of interest (ROIs) included in the Desikan–Killiany–Tourville (DKT) cortical atlas,^31^ and was separately assessed for the left and right hemispheres.

### 2.9 Demographic and biochemical variables

Participants’ age and sex were included as demographic variables. Information on educational level, native language, and history of physician‐diagnosed neuropsychiatric or neurodegenerative disorders was based on self‐reports. Educational level was determined using the International Standard Classification of Education 2011 and was coded as low (lower secondary education or below), middle (upper secondary education to undergraduate university level), and high (postgraduate university study). Native language was coded as German or other. Differential blood cell count measurements (erythrocytes, nucleated erythrocytes, leukocytes, and platelets) were performed at the Central Laboratory of the University Hospital in Bonn, using ethylenediaminetetraacetic acid‐whole blood samples on a hematological analyzer Sysmex XN9000.

### 2.10 Statistical analysis

Descriptive data were expressed as mean ± standard deviation (SD) and counts with proportions for numerical and categorical variables. To compare cognitive scores among the main analytical sample and the subsamples used for brain imaging and gene expression analyses, we used type III analysis of covariance adjusting for age and sex.

To examine the association of miRNA expression with cognitive scores, we followed two complementary methods. First, based on the guilt‐by‐association framework,^32^ we assumed that miRNAs that follow similar expression patterns act jointly to form biologically functional units. We used the weighted gene co‐expression network analysis (WGCNA) method,^33^ to create groups of similarly expressed miRNAs (co‐expression analysis). This allowed us to account for the correlated structure of miRNA expression data, increasing our statistical power for detecting trait‐related miRNAs. Second, we performed a per miRNA analysis, meaning that we directly examined the association of individual miRNAs with cognitive scores. This was done to account for single miRNAs that might be strongly related to cognitive outcomes, but only weakly co‐expressed with other miRNAs, in which case the grouping method would be less effective.

#### 2.10.1 Construction of weighted gene co‐expression network

We clustered miRNAs in co‐expression modules using the WGCNA R package (v.1.70‐3).^33^ We chose a soft thresholding power of seven based on scale‐free topology, and we used the hybrid dynamic tree cut method to identify miRNA modules, with a minimum module size of five. This resulted in 16 modules, which we refer to by randomly assigned colors (green, magenta, turquoise, black, pink, yellow, brown, red, blue, green–yellow, purple, cyan, salmon, light cyan, midnight blue, tan; Figure S1 in supporting information). We examined whether precursors of miRNAs in the same module were also clustered based on genomic position, defined as having precursor sequences located within 10 kb of each other, using data downloaded from miRbase.^34^ We then reduced the expression of all miRNAs in a module to a single value (module expression). Detailed information on the application of the WGCNA algorithm is provided in Methods S2 in supporting information.

#### 2.10.2 Association of module and miRNA expression with cognition

For the co‐expression and per miRNA analyses we examined the association of module and miRNA expression with cognitive scores using multiple linear regression. First, we converted all numeric variables to *z* scores, to permit the comparability of all linear regression coefficients. Then, for each module, each miRNA, and each cognitive score we created a separate model including module or miRNA expression as the independent variable and cognitive score as the dependent variable while adjusting for age and sex (Model 1: *cognitive score* ∼ *miRNA or module expression* + *age* + *sex*). Additionally, we created a second model that included education level and native language as covariates. For crystallized intelligence, however, we did not adjust for native language as German as a native language is a prerequisite for undertaking the test (Model 2: *cognitive score* ∼ *miRNA or module expression* + *age* + *sex* + *educational level* [+ *native language*]). A third model was additionally adjusted for blood cell counts (Model 3: *cognitive score* ∼ *miRNA or module expression* + *age* + *sex + leukocyte count + erythrocyte count + nucleated erythrocyte count + platelet count*). Additionally, we performed a sensitivity analysis by excluding participants who reported a physician‐diagnosed neuropsychiatric or neurodegenerative disorder (i.e., dementia, Parkinson's disease, multiple sclerosis, stroke, schizophrenia), and re‐ran Model 1 in the remaining sample.

The first part of our per miRNA analysis consisted of a replication analysis for a priori selected miRNAs. This included 46 miRNAs associated with cognition in previous similar studies,^12^, ^13^, ^14^, ^15^, ^16^ and 10 miRNAs that were consistently found to be differentially expressed in the blood of patients with AD and mild cognitive impairment (MCI) in a recent meta‐analysis.^17^ An overview of these studies can be found in Table S1 in supporting information. As all of these studies only examined measures of fluid intelligence, for this analysis we only included cognitive scores that reflect fluid intelligence (i.e., global cognition, working memory, episodic verbal memory, processing speed, and executive function). One study examined the association of miRNA expression with cognition using linear regression, as well as comparing expression between two cognitive performance categories.^15^ For this study, we included for replication miRNAs from the linear regression analysis, which presented greater similarity with our method. *P* values for the replication analysis were not corrected for multiple testing.

Beyond the replication analysis, the association of miRNAs with cognition was evaluated without a priori hypotheses and thus we corrected for multiple testing using the Benjamini–Hochberg false discovery rate (FDR) method. Specifically, *p* values were corrected for the number of modules (*n* = 16) for the co‐expression analysis, and for the number of miRNAs (*n* = 415) for the per miRNA analysis.

After identifying modules significantly associated with cognitive scores in the co‐expression analysis, we also identified hub miRNAs in these modules. These were defined as miRNAs significantly associated with the same cognitive score as the module, and highly correlated with module expression (high significance and high membership miRNAs; Methods S2). Here, *p* values were not corrected for multiple testing, as this was only done for the modules already significantly associated with cognitive scores. In all cases, the statistical significance level was set at 0.05 (FDR ≤ 0.05 or *p* value ≤ 0.05).

#### 2.10.3 Interaction and stratification based on age and sex

To examine whether age and sex modified the association of miRNAs with cognitive scores, we used additional linear models, including interaction terms between miRNA expression and age (Model 4: *cognitive score* ∼ *miRNA expression* + *age* + *miRNA* *expression* × *age* + *sex*), or between miRNA expression and sex (Model 5: *cognitive score* ∼ *miRNA expression* + *age* + *sex* + *miRNA expression* × *sex*). For miRNAs already identified as associated with cognition, the statistical significance of interaction terms was determined without adjusting for multiple tests. For the rest of the miRNAs measured in our study, we adjusted for *p* values of interaction terms for multiple testing. For miRNAs with a significant interaction term with either age or sex, we additionally performed a stratification analysis. This was done by splitting our main analytical sample based on median age in older (≥ 54 years) or younger (< 54 years) participants and in men or women. We then ran Model 1 again in these age and sex strata.

#### 2.10.4 Association of cognition‐related miRNAs with brain MRI measures

MiRNAs identified as significantly associated with cognition either in the replication or the hypothesis‐free analyses were evaluated for their association with hippocampal volume, mean cortical thickness, and total brain volume. MiRNAs that were significantly associated with mean cortical thickness were further evaluated for their association with cortical ROI thickness. As for the association of miRNAs with cognitive scores, this was done by first converting numeric variables to *z* scores and then using multivariable linear regression. Brain MRI traits were included as the dependent variable and miRNA expression as the independent variable while adjusting for age and sex. When the dependent variable was hippocampal or total brain volume, we additionally adjusted for estimated total intracranial volume (eTIV), to account for differences in head size (Model: brain *MRI measure* ∼ *miRNA or module expression* + *age* + *sex* [+ *eTIV*]). The results of this analysis were not corrected for multiple testing based on the a priori hypothesis that miRNAs associated with cognition would also be associated with brain structure.

### 2.11 MiRNA and gene expression in tissues and cells

After identifying the miRNAs significantly associated with cognition, we assessed in which tissues they were most highly expressed. For this, we used miRNATissueAtlas2, an online resource that provides data on miRNA expression in 60 human tissues, acquired *post mortem* from six donors.^35^ Using this data, for each miRNA we converted expression across tissues to a *z* score and we calculated the median of miRNA expression for each tissue across donors. From the same resource, we obtained the Tissue Specificity Index, a continuous metric ranging from 0, indicating that a miRNA is expressed across all tissues, to 1, indicating that a miRNA is only expressed in a single tissue. We additionally evaluated whether miRNA expression differed between the brain and other tissues. To do this, we categorized miRNA tissue expression in “Brain” and “Other Tissues” and re‐calculated *z* scores for each miRNA across these two categories. We followed a similar approach to examine miRNA expression in cells. We downloaded miRNA expression data in human cells from an atlas based on the Functional Annotation of the Mammalian Genome (FANTOM5) project.^36^ After the exclusion of cancer cell lines, and cell lines treated with factors that would alter physiological miRNA expression, we averaged expression across multiple samples. Then, for each miRNA, we *z*‐transformed expression across cells to facilitate comparisons.

To identify miRNA target genes that are expressed in the brain, we used data on gene expression in 50 human tissues taken from the Human Protein Atlas.^37^ We filtered the atlas for brain structures and genes that are expressed in these structures, with a cut‐off of normalized transcripts per million (nTPM) >10.

### 2.12 Functional genomics analysis

To determine genes that could be regulated by miRNA modules in vivo, we first obtained putative target genes for each miRNA using the multimir R package (v.1.12.0).^38^ Subsequently, we examined the association between miRNA and target gene expression in our participants using linear regression, adjusting for age, sex, and blood cell counts (Model: *target gene expression* ∼ *miRNA expression* + *age* + *sex* + *erythrocyte count* + *nucleated erythrocyte count* + *leukocyte count* + *platelet count*). We kept for further analysis genes whose expression was negatively associated with the expression of the targeting miRNA. These genes were then used for a pathway enrichment analysis, pooling target genes of miRNAs belonging to the same WGCNA module, and using the Gene Ontology: Biological Processes database and the “clusterProfiler” (v. 3.18.1) R Bioconductor package.^39^ Semantically similar terms were collapsed *post hoc* using the rrvgo (v. 1.2.0) R Bioconductor package,^40^ setting a small similarity threshold of 0.6. For detailed information on the identification of target genes and functional enrichment, refer to Methods S3 in supporting information. We additionally examined the enrichment of target genes among genes identified in a published genome‐wide association study (GWAS) of general cognitive function.^41^ This study identified 859 cognition‐related genes, and gene expression data was available for 544 of those genes in our data. In a similar approach, we examined the overlap of target genes of miRNAs associated with cortical regions among genes identified in a GWAS of regional cortical thickness.^42^ This study identified 29 unique genes associated with mean and regional cortical thickness, for all of which expression was available in our data. The enrichment analysis was done by examining whether the target genes of each miRNA were overrepresented among the genes identified by the GWAS using a hypergeometric test (“hypeR” R Bioconductor package, v.1.10.0). This analysis was performed for individual miRNAs and not per module, to identify the most relevant single miRNAs. The statistical significance level for enrichment analyses was set at 0.05 (FDR ≤ 0.05 or *p* value ≤ 0.05).

### 2.13 miR expression quantitative trait loci analysis

To identify potential miRNA expression quantitative trait loci (miR‐eQTLs), we conducted a genome‐wide miR‐eQTL analysis on 2456 participants of the Rhineland Study, for which both genetic and miRNA expression data were available. Specifically, we used linear regression analysis to evaluate the association between each SNP (independent variable) and expression levels of cognition‐related miRNAs (dependent variable), adjusting for age, sex, and the first 10 genetic principal components. Cis‐SNPs were defined as those located within 1 Mb (1 Mb before the start or 1 Mb after the end) of the mature miRNA sequence).^43^ The genome‐wide significance level for cis miR‐eQTLs was set at *p* value ≤ 5 × 10^−8^. We used the functional mapping and annotation (FUMA) for GWAS platform^44^ to define genomic risk loci, by clumping SNPs in linkage disequilibrium at *r* ^2^ > 0.6. Significant SNPs in relatively high linkage disequilibrium at *r* ^2^ < 0.6 were defined as independent significant SNPs, while significant SNPs in approximate linkage disequilibrium at *r* ^2^ < 0.1 were defined as lead SNPs. Genes were mapped to lead SNPs with positional mapping.

### 2.14 Two‐sample Mendelian randomization

We used a two‐sample Mendelian randomization analysis to examine whether the associations between miRNAs and cognition could be causal. Mendelian randomization uses genetic variants as instrumental variables to infer the causal influence of an exposure on an outcome, based on the assumption that alleles are randomly inherited, therefore simulating a randomized controlled trial.^45^ The analysis was done using the TwoSampleMR R package.^45^ We included as instrumental variables cis‐SNPs that were significant at the *p* value ≤ 1 × 10^−5^ significance level in the miR‐eQTL analysis^43^ and as the exposure to the expression of miRNAs associated with cognition. Cognition was included as the outcome, based on published summary statistics from a GWAS of general cognitive function.^41^ Before the analysis, we clumped SNPs in linkage disequilibrium, defined as *r* ^2^ < 0.001 within a 10 Mb window. We then performed a Mendelian randomization analysis using the Wald ratio test, when only one SNP instrumental variable was available, and the inverse weighted variance and MR Egger methods when multiple SNP instrumental variables were available. We additionally examined for inverse causation by performing the same analysis, but this time, coding cognition as the exposure and miRNA expression as the outcome. To assess the risk of weak instrument bias, we calculated the F statistic for the selected SNP instrumental variables.

All analyses were performed using the R programming environment (v. 4.1.0) unless stated otherwise.

### 2.15 Data availability

The data from the Rhineland Study are not publicly available due to data protection regulations. Access to data can be provided to scientists in accordance with the Rhineland Study's Data Use and Access Policy. Requests for additional information or access to the Rhineland Study's datasets can be sent to RS-DUAC@dzne.de.

## 3 RESULTS

### 3.1 Participant characteristics

In our main analytical sample (cognition dataset, *n* = 2869 participants), the mean age was 55.01 years (SD = 14.13 years, range = 30–95 years), and 56% of participants were women. The subset of participants with complete MRI data was slightly younger and had slightly higher cognitive domain scores (Table 1). However, when adjusted for age and sex, the cognitive domain scores did not significantly differ between the cognition dataset and its subsets (all *p* values > 0.1).

**TABLE 1:** Study participant characteristics.

Variable	Participants with complete cognitive examination and microRNA expression data, *N* = 2869^a^	Participants with complete cognitive examination, microRNA expression, and brain MRI data, *N* = 2045^b^, ^c^	Participants with complete cognitive examination, microRNA expression, and gene expression data, *N* = 2138^c^
Age (years), mean (SD)	55.01 (14.13)	54.31 (13.90)	54.99 (14.18)
Sex, *n* (%)			
Female	1595 (56%)	1149 (56%)	1173 (55%)
Male	1274 (44%)	896 (44%)	965 (45%)
Global cognition (*z* score), mean (SD)^d^	0.01 (0.64)	0.05 (0.62)	0.01 (0.63)
Executive function (*z* score), mean (SD)^d^	−0.03 (0.75)	0.02 (0.73)	−0.02 (0.75)
Working memory (*z* score), mean (SD)^d^	−0.01 (0.72)	0.03 (0.71)	0.00 (0.71)
Ep. verbal memory (*z* score), mean (SD)^d^	0.06 (0.96)	0.11 (0.93)	0.04 (0.96)
Processing speed (*z* score), mean (SD)^d^	0.02 (0.83)	0.06 (0.82)	0.03 (0.83)
Crystalized intelligence (*z* score), mean (SD)^d^	0.02 (0.96)	0.06 (0.93)	0.02 (0.96)
First language, *n* (%)			
German	2682 (94%)	1914 (94%)	2006 (94%)
Other	182 (6.4%)	130 (6.4%)	129 (6.0%)
Education level, *n* (%)			
Low	55 (1.9%)	34 (1.7%)	34 (1.6%)
Middle	1248 (44%)	844 (42%)	928 (44%)
High	1543 (54%)	1152 (57%)	1155 (55%)
Left hippocampal volume (mm^3^), mean (SD)	–	3.89 × 10^3^ (0.46 × 10^3^)	3.88 × 10^3^ (0.46 × 10^3^)
Right hippocampal volume (mm^3^), mean (SD)	–	4.05 × 10^3^ (0.49 × 10^3^)	4.04 × 10^3^ (0.49 × 10^3^)
Total brain volume (mm^3^), mean (SD)	–	1116.40 × 10^3^ (120.73 × 10^3^)	1115.78 × 10^3^ (120.02 × 10^3^)
eTIV (mm^3^), mean (SD)	–	1.56 × 10^6^ (0.16 × 10^6^)	1.56 × 10^6^ (0.16 × 10^6^)
Mean cortical thickness (mm), mean (SD)	–	2.45 (0.09)	2.45 (0.09)

Abbreviations: eTIV, estimated total intracranial volume; MRI, magnetic resonance imaging; SD, standard deviation.

^a^ Participants with data additionally missing for: crystallized intelligence, *n* = 190; blood cell counts, *n* = 152.

^b^ Values have not been adjusted for head size. Participants with data additionally missing for individual imaging markers: cortical thickness: *n* = 17; total brain volume: *n* = 18; hippocampal volume: *n* = 4.

^c^ Subset of participants with complete cognitive examination and microRNA expression data.

^d^ Cognitive examination *z* scores were generated based on summary statistics from the first 5000 participants of the Rhineland Study.

### 3.2 Association of miRNAs and miRNA co‐expression modules with cognition

#### 3.2.1 Replication of miRNAs previously associated with cognition, AD, or MCI

**FIGURE 1:**
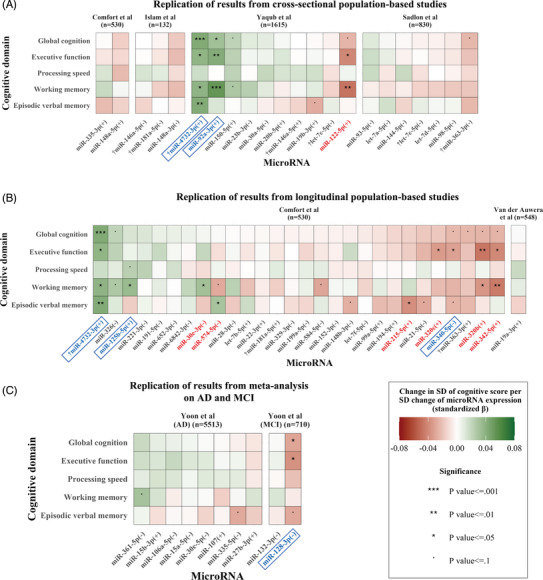
Replication of previous findings on miRNAs associated with cognition, AD, or MCI. Replication of miRNAs that were identified as associated with cognition in previous cross‐sectional population‐based studies^12^, ^13^, ^14^, ^15^, ^16^ (A), one previous longitudinal population‐based study (B),^13^ and a previous meta‐analysis of miRNAs dysregulated in AD and MCI (C).^17^ Details on the studies included can be found in Table S1 in supporting information. The sample sizes of included studies are in parentheses next to the study names. Statistical significance is indicated by the asterisks. MiRNAs that appeared in multiple included studies are marked with “†.” MiRNAs are marked with “+” in parentheses when their higher expression was associated with better cognitive function in included studies, and with “–” in the opposite case. For the AD and MCI meta‐analysis, “+” indicates higher expression of the miRNA in controls compared to patients. MiRNAs that were significantly associated with cognition with a similar direction in the included studies and in our data have been annotated with blue color and are outlined with a blue box. Annotated with red color are miRNAs that were significantly associated with cognition in our study, but in an opposite direction compared to included studies. AD, Alzheimer's disease; MCI, mild cognitive impairment; miRNA, microRNA; SD, standard deviation.

Among the 46 miRNAs associated with cognition in previous population‐based studies, miR‐340‐5p, miR‐125b‐5p, mir‐4732‐3p, and miR‐92a‐3p were associated with at least one cognitive score in the same direction in our per miRNA analysis. Of note, miR‐4732‐3p had been found in two of the previous studies^12^, ^13^ and, among these 46 miRNAs, it had the strongest association with cognition in our data. Conversely, seven miRNAs were significantly associated with cognitive scores in the opposite direction in our data, compared to previous studies. Six of these miRNAs, miR‐30e‐3p, miR‐342‐5p, miR‐574‐5p, miR‐320c, miR‐215‐5p, miR‐320b, miR‐122‐5p, came from the same study by Comfort et al.,^13^ which had included older (median age: 71 years) and only male participants. The seventh miRNA, miR‐122‐5p, had been identified in a study by Yaqub et al.,^12^ which also included older participants (median age: 70.3 years) compared to our study (Figure 1A,B). To examine whether these inconsistent findings were due to the age or sex differences in the examined populations, we included the six miRNAs from Comfort et al. in a sex‐stratified analysis, and all seven miRNAs in an age‐stratified analysis (shown in Section 3.2.5). Notably, both Yaqub et al.^12^ and Comfort et al.^13^ examined miRNAs derived from plasma samples, as opposed to our study, which measured miRNAs in whole blood samples (Table S1).

Among the 10 miRNAs differentially expressed in AD and MCI,^17^ higher miR‐128‐3p expression had been previously found higher in MCI patients and was associated with worse global cognition, executive function, and episodic verbal memory performance in our data (Figure 1C).

#### 3.2.2 Hypothesis‐free co‐expression analysis

**FIGURE 2:**
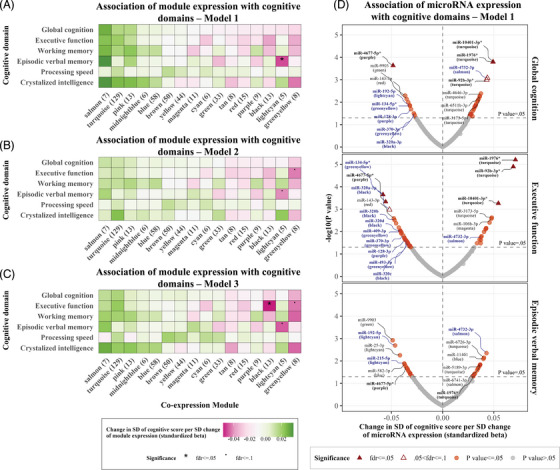
Hypothesis‐free association of miRNA and module expression with cognition. A–C, Linear regression coefficients and significance levels for the association between co‐expression modules and cognitive scores. Shown are Model 1 (A) which was adjusted for age and sex; Model 2 (B) which was adjusted for age, sex, first (native) language, and educational level; and Model 3, which was adjusted for age, sex, and blood cell counts. Modules are named after colors, and numbers in parentheses indicate the number of miRNAs within each module. Significance was determined after correcting for multiple comparisons using the FDR method. (D) Volcano plots of the per miRNA association of all miRNAs measured in our study with global cognition, executive function, and episodic verbal memory scores. The *p* value shown in the y axis has not been corrected for multiple testing. Top miRNAs for each score are annotated and co‐expression modules are shown in parentheses. MiRNAs that reached statistical significance for at least one phenotype, after multiple testing correction (FDR ≤ 0.05), are shown with triangles and have been marked with an asterisk (*). Hub miRNAs are annotated with blue color. FDR, false discovery rate; miRNA, microRNA; SD, standard deviation.

Beyond the replication of previous studies, we performed a hypothesis‐free analysis to identify novel miRNA co‐expression modules and hub miRNAs associated with cognition. Using WGCNA, we identified 16 miRNA co‐expression modules with a size ranging from 5 to 129 miRNAs. Modules were named after randomly assigned colors (Figure S1 and Table S2 in supporting information). The miRNAs grouped in the same module had 41 unique precursor sequences with genomic locations within 10 kb of each other. Conversely, another 45 precursor sequences were located within 10 kb of each other, but the corresponding mature miRNAs were grouped in different co‐expression modules (Table S2). Adjusting for age and sex, and after correction for multiple testing, higher expression of only one of these modules (light cyan) was significantly associated with worse episodic verbal memory (standardized ß: −0.048, 95% confidence interval [CI]: −0.078 to −0.019, FDR‐corrected *p* value: 0.021, adjusted *R* ^2^: 0.35). Additionally, we observed trends toward the association of lower expression of two modules (green–yellow, black) and higher expression of three modules (salmon, turquoise, and pink) with better performance across multiple scores. However, none of these associations were significant after multiple testing corrections (Figure 2A and Table S3 in supporting information). When additionally adjusting for educational level and first language, increased expression of the green–yellow module became significantly associated with worse executive function (standardized ß: −0.045, 95% CI: −0.075 to −0.016, FDR‐corrected *p* value: 0.043, adjusted *R* ^2^: 0.36). With the same adjustment, higher expression of the light cyan module remained significantly associated with worse episodic verbal memory, yet the association was slightly weakened (Figure 2B and Table S3). In the model adjusted for blood cell counts, increased expression of the black module also became significantly associated with worse executive function (standardized ß: −0.053, 95% CI: −0.083 to −0.023, FDR‐corrected *p* value: 0.022, adjusted *R* ^2^: 0.30), while associations remained similar but slightly weaker for the light cyan and green–yellow modules (Figure 2C and Table S3). Based on these findings, we decided to retain the green–yellow, light cyan, and black modules for further analyses, as they demonstrated the strongest and most consistent associations with cognitive function.

We then identified hub miRNAs in these two modules, defined as miRNAs strongly correlated with module expression, while being significantly associated with the same cognitive domains as the modules (i.e., executive function for the green–yellow and black module, episodic verbal memory for the light cyan module). In the model adjusted for age and sex, these conditions were fulfilled for four miRNAs from the green–yellow module, miR‐134‐5p, miR‐409‐3p, miR‐370‐3p, and miR‐493‐3p; for two miRNAs from the light cyan module, miR‐215‐5p and miR‐192‐5p; and for four miRNAs from the black module, miR‐320a‐3p, miR‐320b, miR‐320c, and miR‐320d (Figure 2D and Table S4 in supporting information). The four miRNAs from the black module are all members of the mir‐320 gene family.^34^

#### 3.2.3 Hypothesis‐free per miRNA analysis

For the next step of the hypothesis‐free analysis, we individually examined all miRNAs measured in our study (*n* = 415) in relation to cognitive function, regardless of the co‐expression module. Adjusting for age and sex, we identified four miRNAs, miR‐92b‐3p, miR‐1976, miR‐10401‐3p, and miR‐4677‐5p, which were significantly associated with executive function after multiple testing corrections. The same miRNAs were significantly associated with global cognition, with the exception of miR‐92b‐3p, which was associated with global cognition at borderline significance (Figure 2D and Table S4). Results changed only slightly when additionally adjusting for education and native language, or for blood cell counts (Figure S2 in supporting information). Of note, miR‐92b‐3p is closely related to miR‐92a‐3p, which was identified in our replication analysis (Section 3.2.1). These two miRNAs belong to the same family (mir‐25 family) and share highly similar mature sequences.^34^ Despite that, their expression levels were only moderately correlated in our data (Pearson correlation coefficient: 0.37, 95% CI: 0.34 to 0.40).

**FIGURE 3:**
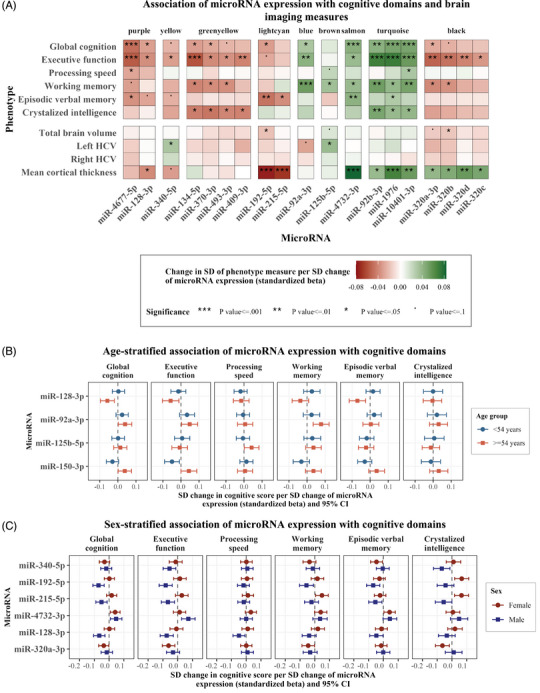
Association of miRNA expression with cognitive scores and brain imaging measures. The heatmap (A) shows the association of miRNA expression with cognitive scores and brain imaging measures. Included were miRNAs identified as associated with cognition in our literature replication, co‐expression, and per miRNA analyses. The association of some miRNAs with cognitive domain scores was modified by sex and age. This is illustrated in (B) for age and (C) for sex, which show the standardized regression betas and 95% confidence intervals for the age‐ and sex‐stratified association of these miRNAs with each cognitive score. Linear regression analyses were based on Model 1, which included as covariates age and sex, except for the sex‐stratified analysis which was controlled only for age. CI, confidence interval; FDR, false discovery rate; HCV, hippocampal volume; miRNA, microRNA; SD, standard deviation.

Thus, combining the results from our literature replication, as well as our co‐expression and per miRNA hypothesis‐free analyses, we detected a signature of five previously identified (miR‐340‐5p, miR‐125b‐5p, mir‐4732‐3p, miR‐92a‐3p, and miR‐128‐3p) and 14 novel miRNAs (miR‐192‐5p, miR‐134‐5p, miR‐370‐3p, miR‐409‐3p, miR‐493‐3p, miR‐215‐5p, miR‐4677‐5p, miR‐10401‐3p, miR‐1976, miR‐92b‐3p, miR‐320a‐3p, miR‐320b, miR‐320c, and miR‐320d) that were strongly related to cognition. Notably, while significant associations were stronger for the executive function and episodic verbal memory scores, these 19 miRNAs were nominally associated with most other cognitive scores as well (Figure 3A and Table S4). Median values and distribution of the expression (normalized counts) of the 19 miRNAs can be found in Figure S3 in supporting information.

#### 3.2.4 Neuropsychiatric and neurodegenerative disorder sensitivity analysis

To ensure that our results were not confounded by dysregulation of miRNA expression due to neuropsychiatric or neurodegenerative disorders, we re‐ran the model adjusted for age and sex after excluding participants with a self‐reported physician diagnosis of dementia (*n* = 3), Parkinson's disease (*n* = 8), multiple sclerosis (*n* = 13), stroke (*n* = 48), and schizophrenia (*n* = 2). This sensitivity analysis resulted in only slight differences in linear regression coefficients for the 16 co‐expression modules and the 19 cognition‐related miRNAs, without affecting statistical significance (Figure S4 in supporting information).

#### 3.2.5 Age and sex as effect modifiers

Next, we examined whether the association of the identified miRNAs with cognition was modified by age or sex. We detected significant positive interaction terms with age for the associations of miR‐128‐5p with episodic verbal memory and global cognition, miR‐125b‐5p with executive function and crystallized intelligence, and miR‐92a‐3p with working memory (Table S5 in supporting information). An age‐stratified analysis confirmed that the association of these miRNAs with cognitive scores was stronger in the older age group (≥ 54 years; Figure 3B). Additionally, a significant sex interaction was observed for the association of miR‐192‐5p and miR‐215‐5p (light cyan module) with multiple cognitive domains, miR‐4732‐3p with executive function, as well as miR‐340‐5p and miR‐320a‐3p with crystallized intelligence (Table S6 in supporting information). A sex‐stratified analysis showed that these miRNAs were more strongly associated with most cognitive domains in men, with the exception of miR‐320a‐3p, which showed stronger associations in women. The effect modification was especially pronounced for miR‐215‐5p. Higher expression of this miRNA was associated with worse working memory and crystallized intelligence performance in the total sample and among men, but with better performance in these domains among women (Figure 3C).

As some miRNAs might be associated with cognition only in younger or older participants, we applied the same analysis to all 415 miRNAs identified in our study. After adjustment for multiple testing, we detected a significant positive interaction term for the association of miR‐150‐3p with executive function (Table S5). Age stratification showed that this miRNA was associated with worse cognition in younger participants, but better cognition in older participants (Figure 3B).

Seven miRNAs, including the abovementioned miR‐215‐5p, were inconsistently associated with cognition in our study and in previous studies by Comfort et al. and Yaqub et al.^12^, ^13^ (Section 3.2.1). As these previous studies included older participants compared to ours, the inconsistent associations of miRNAs with cognition might have been driven by age‐dependent effects. Age‐stratified analysis for these miRNAs partially confirmed this assumption. Specifically, for four miRNAs (miR‐342‐5p, miR‐320b, miR‐320c, and miR‐574‐5p), the inconsistent association with cognition was stronger in younger participants and tended toward the null in older participants (Figure S5A in supporting information). Similarly, the study by Comfort et al. included only male participants. For four of the miRNAs identified in this study (miR‐342‐5p, miR‐320b, miR‐320c, and miR‐30e‐3p) the inconsistent association with cognition was stronger in women and tended toward the null in men (Figure S5B). However, the assumption of sex driving the inconsistent association with cognition was not confirmed for miR‐215‐5p. As mentioned above, higher miR‐215‐5p expression was associated with much worse cognitive function among men in our study, but with better cognitive function among men in Comfort et al.

### 3.3 Association of cognition‐related miRNAs with brain MRI measures

Next, we assessed whether the expression of the 19 miRNAs identified as associated with cognition was also associated with mean cortical thickness, hippocampal volume, and total brain volume. Higher expression of miR‐128‐3p, miR‐215‐5p, and miR‐192‐5p was significantly associated with a thinner cortex, while higher miR‐192‐5p and miR‐320b expression was additionally associated with smaller total brain volume. On the contrary, higher expression of eight miRNAs, miR‐4732‐3p, miR‐92b‐3p, miR‐1976, miR‐10401‐3p, miR‐320a‐3p, miR‐320b, miR‐320c, and miR‐320d, was associated with a thicker cortex. Moreover, higher miR‐125b‐5p expression was associated with larger left hippocampal volume, which was consistent with its association with better cognitive function. Surprisingly, although miR‐340‐5p expression was associated with worse cognitive function, it was also associated with larger left hippocampal volume and, at borderline significance, a thinner cortex (Figure 3A and Table S7 in supporting information).

**FIGURE 4:**
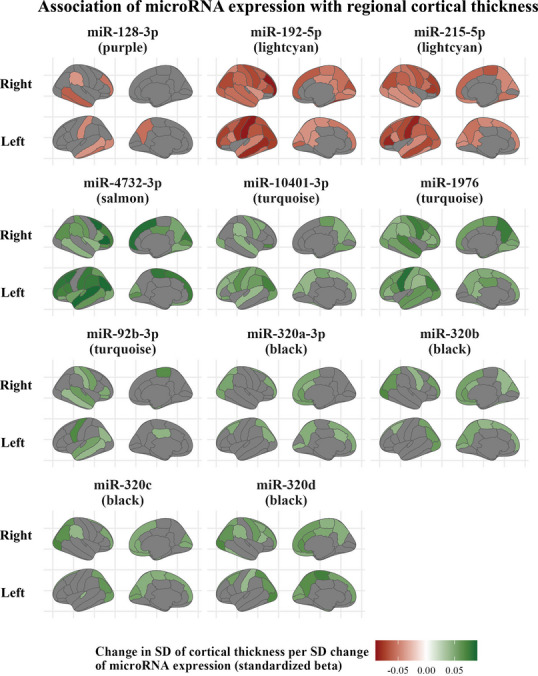
Region‐specific association between miRNA expression and brain cortical thickness. Regions of interest have been mapped based on the Desikan–Killiany cortical atlas. The color indicates the standardized *β* for the regions in which miRNA expression was significantly associated with cortical thickness. The analysis was performed only for miRNAs that were significantly associated with cognition and mean cortical thickness across both hemispheres. miRNA, microRNA; SD, standard deviation.

ROI‐specific analysis showed that the association of miRNAs with cortical thickness was overall strongest in the left hemisphere, and followed similar patterns for miRNAs in the same module. Specifically, the associations were strongest in the left middle temporal and postcentral gyri for light cyan module miRNAs (miR‐215‐5p, miR‐192‐5p), and in the left precentral and superior temporal gyri for turquoise model miRNAs (miR‐10401‐3p, miR‐1976, and miR‐92b‐3p). For miR‐4732‐3p (salmon module), the associations were strongest bilaterally in the precentral and superior temporal regions, and in the right paracentral region. The association of miR‐128‐3p (purple module) with cortical thickness was overall weaker and localized in the right middle and inferior temporal regions. The associations were also weaker for black module miRNAs (miR‐320a‐3p, miR‐320b, miR‐320c, and miR‐320d), and localized bilaterally in the inferior parietal as well as the right lateral occipital regions (Figure 4 and Table S8 in supporting information).

Last, it is worth mentioning that the expression of miR‐1976, miR‐92b‐3p, miR‐128‐3p, miR‐192‐5p, miR‐320b, miR‐320c, and miR‐320d was significantly or borderline significantly associated with the thickness of the entorhinal cortex (Table S8).

### 3.4 MiRNA expression in tissues and cells

**FIGURE 5:**
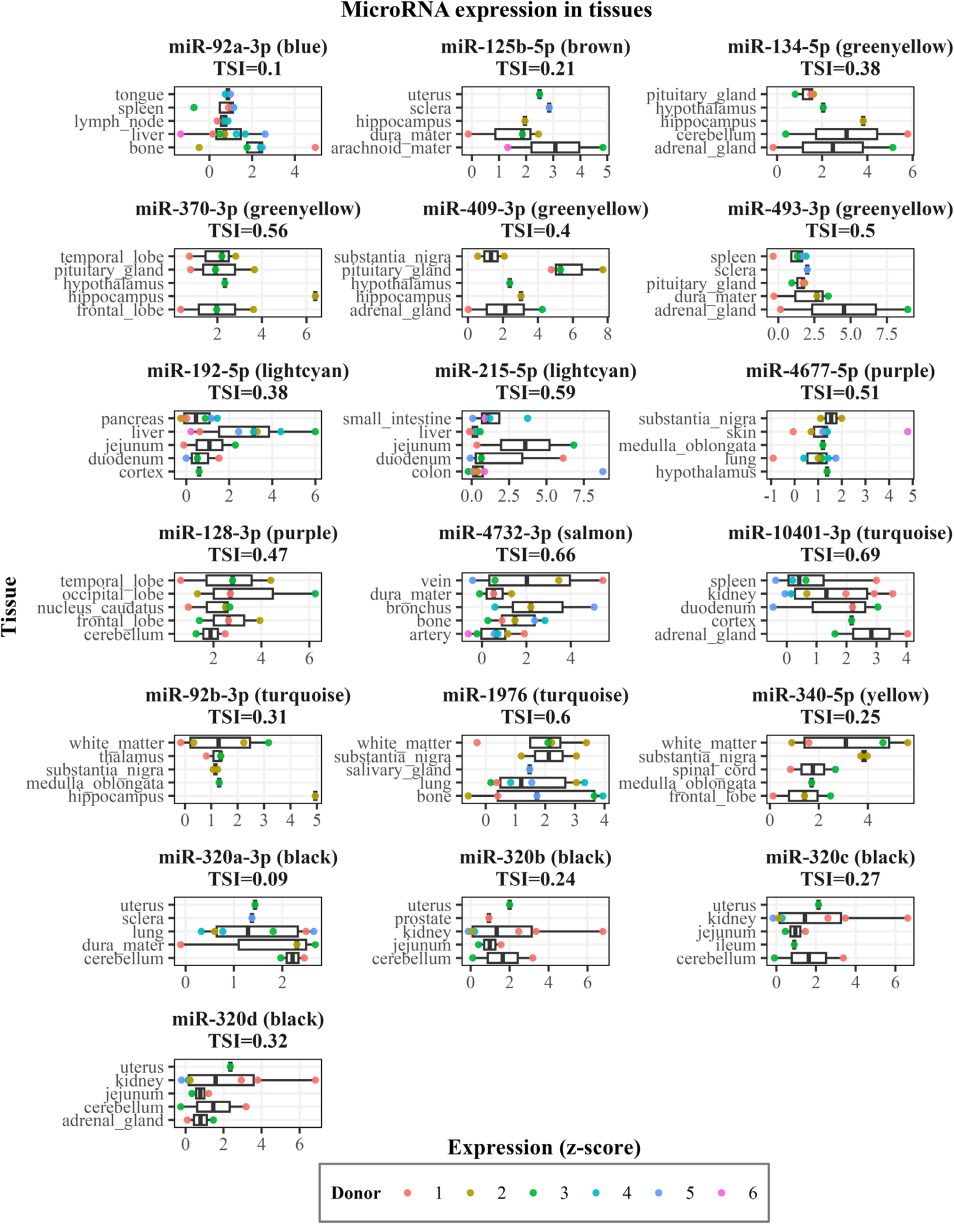
Tissue expression analysis of cognition‐related miRNAs. Boxplots of tissue expression of miRNAs associated with cognition. Data were obtained from the miRNA Tissue Atlas,^35^ based on *post mortem* samples taken from six human donors (here indicated by the colored dots). The module each miRNA belongs to is shown in parentheses. The top five tissues for each miRNA, based on median miRNA expression, are shown. Note that, to allow for better comparison of relative expression in tissues for each miRNA, the scale of the *x* axis (expression *z* score) varies. miRNA, microRNA; TSI, tissue specificity index.

Using miRNATissueAtlas data, we determined that the majority of the 19 cognition‐related miRNAs were expressed most abundantly across brain areas, such as the hippocampus, medulla oblongata, cerebellum, and cortex. However, miRNAs from the light cyan, blue, and salmon modules were exceptions, as they were expressed most abundantly in other tissues. In addition to being associated with cortical thickness, miR‐128‐3p, miR‐10401‐3p, and miR‐192‐5p were also abundantly expressed in the cortex. Similarly, miR‐125b‐5p, which was associated with working memory and hippocampal volume, was also highly expressed in the hippocampus. The Tissue Specificity Index of the 19 miRNAs ranged from 0.09 (miR‐320a‐3p) to 0.69 (miR‐10401‐3p), indicating that none of them was very specific to a single tissue (Figure 5). When grouping tissue expression between the brain and other tissues, we observed that expression was considerably higher in the brain for miR‐125b‐5p, miR‐134‐5p, miR‐370‐3p, miR‐409‐3p, miR‐128‐3p, miR‐92b‐3p, miR‐1976, and miR‐340‐5p. Conversely, miR‐192‐5p, miR‐215‐5p, miR‐320b, miR‐320c, and miR‐320d tended to be more expressed in other tissues compared to the brain (Figure S6 in supporting information).

When evaluating the expression of miRNAs in cell lines, we observed that miR‐125b‐5p, miR‐128‐3p, and miR‐340‐5p expression were markedly higher in neural stem cells. Conversely, miR‐1976, miR‐340‐5p, miR‐4732‐3p, miR‐92a‐3p, miR‐92b‐3p, miR‐320b, and miR‐320c were highly expressed in white blood cells. Cell expression data for miR‐10401‐3p was not available (Figure S7 in supporting information).

### 3.5 Functional genomics analysis

#### 3.5.1 Identification of miRNA target genes

To better understand the function of the cognition‐related miRNAs, we leveraged gene expression data, available in a subset of our study participants. In total, the 19 cognition‐related miRNAs had 9445 unique putative target genes, and for 2911 of them expression of the targeting miRNA was negatively associated with gene expression. The close biological relation between miR‐92a‐3p and miR‐92b‐3p was corroborated by their relatively high number of shared negatively associated target genes (*n* = 382 genes). Notably, these two miRNAs also shared a high number of negatively associated target genes with miR‐128‐3p (*n* = 97). Similarly, the miRNAs from the black module, which belong to the mir‐320 family, shared several negatively associated target genes with each other (*n* = 329 genes) and with miR‐340‐5p (*n* = 95 genes). Beyond that, the 19 miRNAs had relatively few shared target genes (Figure S8 and Table S9 in supporting information). Using data from the Human Protein Atlas,^37^ we determined that, from the 2911 negatively associated target genes, 2269 (78%) were expressed in brain tissues.

#### 3.5.2 Pathway enrichment analysis of miRNA target genes

**FIGURE 6:**
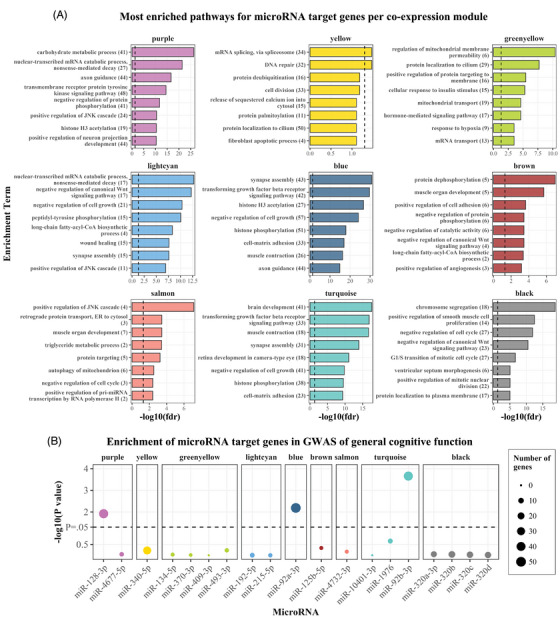
Function genomics analysis of cognition‐related miRNAs. A, Results of Gene Ontology: Biological Processes enrichment analysis of negatively associated target genes of the miRNAs in each co‐expression module. The top eight most enriched terms (based on *p* values) are shown. The numbers in parentheses indicate the number of target genes in each enriched term. *p* values have been corrected for multiple testing using the FDR method, with the dashed line indicating the FDR = 0.05 threshold. Note that, to allow for better comparison of relative enrichment in each module, the scale of the *x* axis (negative logarithm of FDR) varies. B, Overlap and overrepresentation (determined with a hypergeometric test) of negatively associated target genes of miRNA in a GWAS of global cognitive function.^41^ FDR, false discovery rate; GWAS, genome‐wide association study; miRNA, microRNA.

We then performed a Gene Ontology functional enrichment analysis of the negatively associated target genes per WGCNA module. This highlighted the distinct biological functions performed by each module. For example, the green–yellow module was highly enriched for “mitochondrial membrane permeability” and “mitochondrial transport”; the light cyan module was highly enriched for “Wnt signaling,” “cell growth,” and “wound healing”; while the turquoise module was highly enriched for “brain development.” Notably, pathways highly relevant to cognition were jointly enriched for several modules. This included “axon guidance,” “dendrite development,” “neurogenesis,” and “synapse assembly” for the turquoise, purple, light cyan, and blue modules. Similarly, “transforming growth factor beta (TGF‐β) signaling” was among the top results for the turquoise, brown, purple, light cyan, blue, and black modules, while “memory” was enriched for the turquoise, purple, green–yellow, and blue modules. Moreover, “response to estradiol” was enriched for the light cyan module (Figure 6A and Table S10 in supporting information). When filtering for negatively associated target genes that are expressed in brain tissues, the results of the enrichment analysis remained highly similar (Figure S9 in supporting information).

#### 3.5.3 MiRNA target genes in relation to general cognitive function and cortical thickness

To further validate the involvement of the 19 identified miRNAs in cognition, we tested the overlap between miRNA target genes and genes identified by a recent GWAS of general cognitive function.^41^ Notably, targets of miR‐92b‐3p, miR‐92a‐3p, and miR‐128‐3p were significantly overrepresented among the genes identified by the GWAS (Figure 6B and Table S11 in supporting information). Likewise, we examined the overlap between target genes of miRNAs associated with regional cortical thickness, and genes associated with cortical thickness in the same regions or mean cortical thickness in a recent GWAS.^42^ For regional thickness, no overlaps were found, perhaps due to the small number of genes associated with cortical thickness in this GWAS (*n* = 29). The only overlap was found for *AGBL5*, a target gene of miR‐92b‐3p, which was associated with mean cortical thickness in the GWAS.

### 3.6 Genetic variants influencing miRNA expression (miR‐eQTLs)

As the next step in our analysis, we identified genetic variants that influence the expression of miRNAs associated with cognition. We determined that the expression of miR‐125b‐5p, miR‐134‐5p, miR‐370‐3p, miR‐409‐3p, and miR‐493‐3p was influenced by a total of 39 unique independent lead SNPs, 34 and 5 of which were in cis and trans, respectively. Conversely, miR‐215‐5p, miR‐1976, miR‐10401‐3p, miR‐320a‐3p, and miR‐320c expression was only influenced by nine independent lead trans‐SNPs (Figure S10 and Table S12 in supporting information). The strongest genetic influence was found for miR‐125b‐5p, whose expression was influenced by 16 independent significant lead SNPs, with top lead cis‐SNP rs2370750 (standardized ß: 1.10, 95% CI: −1.03 to 1.17, *p* value: 7 × 10^−214^). Of note, we detected a common genetic influence on the miRNAs from the green–yellow module, miR‐134‐5p, miR‐370‐3p, miR‐409‐3p, and miR‐493‐3p. Eight independent significant lead SNPs influenced the expression of two or more of these miRNAs, which are closely located within < 200 kb of each other at the 14q32 genomic locus (Table S2).

### 3.7 Mendelian randomization analysis

Using cis‐miR‐eQTLs as genetic instruments, we performed a Mendelian randomization analysis to detect potentially causal associations between miRNAs and cognition. However, this analysis returned no significant results (all *p* values > 0.1). Similarly, we did not find any evidence of reverse causation, that is, of cognition influencing miRNA expression. The *F* statistic ranged between 25 (when miR‐4732‐3p was coded as exposure) and 371 (when miR‐125b‐5p was coded as exposure), indicating that there is a low risk of weak instrument bias (Table S13 in supporting information).

## 4 DISCUSSION

In the present study, we identified 19 miRNAs associated with cognitive domains in a large, general population‐based cohort with a wide age range. Importantly, five of these miRNAs were identified by reproducing the results of previous studies. Our subsequent analyses using individual‐level brain imaging, genomics, and gene expression data enabled a detailed characterization of identified miRNAs, providing information on the mechanisms underlying their relation to cognition. Some miRNAs were associated with cortical thickness and hippocampal volume, while most were highly expressed in brain tissues and regulated key neuronal processes. Taken together, our large sample size, inclusion of previous studies, and analysis of multimodal data increase the robustness of our findings. Additionally, our results highlight the potential of certain miRNAs as biomarkers of cognitive dysfunction.

MiRNAs associated with different cognitive domains or cortical regions could be investigated as blood‐based biomarkers for disorders that preferentially affect these domains or regions, such as AD for memory and the temporal lobe, or frontotemporal dementia for executive function and the frontal lobe.^46^ For example, miR‐128‐3p, which was upregulated in patients with MCI in a past meta‐analysis, was also associated with worse cognition in our study. In addition to MCI, miR‐128‐3p has also been dysregulated in AD,^17^ while suppressing its expression has been found to protect against AD pathology and memory loss in mice.^47^ Moreover, we showed that it was associated with thickness in the temporal lobe and entorhinal cortex, which are affected very early in the pathophysiology of AD.^48^ Early upregulation of this miRNA could indicate accelerated neurodegeneration, leading to brain atrophy and cognitive dysfunction. This would make it an ideal target for detecting and preventing AD‐related cognitive dysfunction. However, contrary to the findings of our and other studies, a previous population‐based study found that miR‐128‐3p was downregulated in participants with worse cognition.^15^ Thus, further investigations are needed to determine its exact association with cognition.

Several of the identified miRNAs were associated with cortical thickness in regions involved in various cognitive processes. This could indicate that their relation with cognition is mediated by the modulation of brain structure. For example, miRNAs were strongly associated with cortical thickness in the precentral, superior temporal (miR‐10401‐3p, miR‐1976, miR‐92b‐3p, miR‐4732‐3p), postcentral, and middle temporal regions (miR‐215‐5p, miR‐192‐5p, miR‐128‐3p). The superior and middle temporal regions are implicated in multiple cognitive tasks, including audiovisual processing and integration,^49^ declarative memory, and language.^50^ The localization in the left postcentral and precentral gyri (primary somatosensory and motor cortex) might implicate these miRNAs in normal, non‐neurodegenerative brain aging, as age‐related atrophy is increased in these areas.^30^ Moreover, recent evidence supports the involvement of the sensorimotor region in higher cognitive functions, such as attention and learning for the primary motor cortex,^51^ or associative episodic memory for the somatosensory cortex.^52^ Last, miR‐340‐5p and miR‐125b‐5p were associated with the volume of the hippocampus, perhaps the most established region in relation to memory and AD.^53^

Our functional genomics analysis determined that most of the 19 miRNAs jointly regulate processes intrinsically related to cognition, such as memory, axon guidance, dendrite development, neurogenesis, and synapse assembly. In addition, miRNAs from five modules jointly regulated TGF‐β signaling, which plays an important role in neuronal development and neuroprotection in neurodegeneration.^54^ Of note, this included miR‐192‐5p, which has been found to directly affect cognitive function through the modulation of TGF‐β signaling in a mouse model of depression.^55^ Despite this overlap in enrichment results, the miRNAs in each module shared few common target genes, suggesting that they are involved in different, complementary stages of the abovementioned processes. These findings point toward possible interventions for the prevention or treatment of cognitive dysfunction. For example, miR‐192‐5p and miR‐215‐5p were associated with temporal lobe thickness and episodic verbal memory. The target genes of these miRNAs were related to Wnt signaling, especially known for its involvement in synaptic plasticity and AD pathology.^56^ This interaction with Wnt signaling, which for miR‐192‐5p has also been observed experimentally,^57^ could be further investigated for preventing cortical atrophy and cognitive dysfunction in aging or AD. Furthermore, the functional genomics analysis highlighted the importance of miR‐92a‐3p, miR‐92b‐3p, and miR‐128‐3p, as a significant proportion of their target genes have been linked to cognition in a previous GWAS.^41^ Moreover, we determined that the gene *AGBL5*, targeted by miR‐92b‐3p, has been linked to mean cortical thickness. This gene encodes for a metallocarboxypeptidase involved in protein deglutamylation. While mutations in *AGBL5* are most known for causing retinitis pigmentosa, it has also been suggested that they lead to intellectual disability.^58^

Some of the identified miRNAs have been causally linked to cognition or brain health in past experimental studies. Among them, miR‐134‐5p has been most extensively studied as a regulator of synaptic plasticity, dendritogenesis, long‐term potentiation, and neuronal injury.^59^, ^60^, ^61^, ^62^, ^63^ Dysregulation of these processes by miR‐134‐5p can lead to cognitive deficits.^61^, ^64^, ^65^ Similarly, miR‐409‐3p has been found to induce cognitive deficits through impairment of neuronal viability,^66^ while miR‐192‐5p mediates the effects on cognitive function of exercise,^67^ and as mentioned above, depression through TGF‐β.^55^ Moreover, miR‐340‐5p, miR‐92a‐3p, and miR‐92b‐3p have been found to exert neuroprotective effects,^68^, ^69^, ^70^ while miR‐493‐3p, miR‐1976, and miR‐125b‐5p worsen the neurotoxic effects of stroke,^71^ Parkinson's disease,^72^ and AD.^73^ Notably, miR‐134‐5p, miR‐370‐3p, miR‐409‐3p, and miR‐493‐3p were clustered nearby in the 14q32 genomic region, and their expression was jointly influenced by a few genetic variants. The 14q32 region contains one of the largest miRNA clusters in the genome and has been implicated in various cancers, but also neuronal processes like dendritogenesis and axonal maintenance.^74^

The abovementioned studies suggest causal influences of identified miRNAs on cognition, and this is partially supported by our findings, which showed that they are related to brain structure and regulate neuronal processes. In contrast, our Mendelian randomization analysis was not able to detect such influences. However, it should be noted that, perhaps due to insufficient sample size, we could not obtain genetic instruments for eight miRNAs (miR‐192‐5p, miR‐215‐5p, miR‐4677‐5p, miR‐10401‐3p, miR‐92b‐3p, miR‐340‐5p, miR‐92a‐3p, and miR‐128‐3p). When investigating reverse causation, we found no influence of cognition on these miRNAs. Therefore, we cannot exclude the existence of causal effects. Future investigations on causality could focus on these eight miRNAs, and especially miR‐128‐3p, which seemed to originate from the brain, regulated a large number of cognition‐related genes, and has been linked to MCI.^17^

Some of our findings were unexpected and suggest novel research directions regarding the relation of miRNAs to brain function. We identified seven miRNAs that were associated with cognition in opposite directions in our data compared to two previous studies.^12^, ^13^ We showed that this could be due to the differences in the demographics of examined populations. The difference could also be due to the different biomaterials used. MiRNA composition of whole blood, which was used in our study, has been shown to differ from that of plasma samples, which were used in the two previous studies.^16^ Thus, further validation is needed to determine the precise relation of these miRNAs with cognition. Moreover, four miRNAs (miR‐128‐3p, miR‐92a‐3p, miR‐125b‐5p, and miR‐150‐3p) showed a different association with cognition in older compared to younger participants, which could be due to mechanisms of aging or neurodegeneration. For example, all four miRNAs have been implicated in AD pathogenesis.^47^, ^75^, ^76^, ^77^ Similarly, four miRNAs (miR‐215‐5p, miR‐192‐5p, miR‐4732‐3p, and miR‐340‐5p) were associated with cognitive domains in a sex‐dependent manner. This could be explained by an interaction of these miRNAs with sex hormone signaling, known to impact cognition.^78^ In support of this, our functional enrichment results showed that miR‐215‐5p and miR‐192‐5p might regulate cellular responses to estradiol. For miR‐192‐5p, an interaction with estrogen receptors has also been observed experimentally.^79^ Furthermore, we surprisingly observed that mir‐320 family miRNAs were associated with worse cognition, a smaller brain overall, but also a thicker cortex. In a previous work by our group, these miRNAs were associated with arterial dysfunction and increased white matter hyperintensities.^80^ Thus, they might lead to worse cognition through disruptions of white matter integrity, leading to overall brain atrophy, but have a different function in the cortex. Similarly, higher miR‐340‐5p expression was associated with worse cognition and a thinner cortex. However, it was paradoxically associated with a larger hippocampus and has known neuroprotective effects.^81^ It would be interesting to see if future studies can replicate and disentangle these conflicting findings, which demonstrate the pleiotropic functions of miRNAs.

Our study has both strengths and limitations. As mentioned, our large sample size, wide age range, and inclusion of previous similar studies increase the reliability of our findings. Moreover, we were able to comprehensively characterize miRNAs of interest and draw conclusions as to their function, by combining an extensive neuropsychological test battery, individual‐level gene expression and genomics data, detailed brain imaging measures, and publicly available resources. However, our study is not without drawbacks. Our use of WGCNA is based on the assumption that similarly expressed miRNAs have shared biological functions, a principle that has been previously contested.^82^ Furthermore, as is common in population‐based studies, our study participants were all residents of the same geographical region, limiting the generalizability of our findings. Another limitation is that, while our study focuses on brain function and structure, measured miRNAs and gene transcripts might have originated from blood cells. To account for that, we examined the tissue and cell expression of identified miRNAs and their target genes and considered blood cell counts as confounders. However, given our data and study design, it was not possible to precisely determine the source of RNA transcripts. Last, comparing our results to previous similar studies, there was a relatively small overlap and even some conflicting findings. These inconsistencies could be due to the differences in biological samples and miRNA quantification techniques used. This illustrates the necessity for the validation of study results and the standardization of miRNA sampling and measurement methods.

In conclusion, here we identified a signature of five previously identified and 14 novel miRNAs associated with cognitive function across different domains. Some of these miRNAs were concurrently related to cortical thickness and hippocampal volume, suggesting that they regulate cognitive processes through modulation of brain structure. Our functional genomics analysis demonstrated the importance of the identified miRNAs for a number of neuronal functions and emphasized miR‐92b‐3p and miR‐128‐3p, which were associated with cognition and cortical thickness, regulated cognition‐related genes, and were abundant in the brain. In the future, the identified miRNAs could be leveraged to develop signatures for the early detection of dementia in clinical or research settings. Moreover, although we were not able to detect causal associations, some miRNAs might still impact cognition and could be investigated for the prevention of cognitive dysfunction due to aging or neurodegeneration.

## CONFLICT OF INTEREST STATEMENT

The authors declare no conflicts of interest. Author disclosures are available in the supporting information.

## CONSENT STATEMENT

We obtained written informed consent from all participants of the present study in accordance with the Declaration of Helsinki.

## Supporting information

Supporting Information

Tables S2–S13

ICMJE Disclosure Forms
