# Supplementary material for: Blood‐derived microRNAs are related to cognitive domains in the general population
Source: Alzheimers Dement. 2024 Aug 29;20(10):7138–59. doi: 10.1002/alz.14197 (PMC11485070; doi:10.1002/alz.14197)
Supplement: Supplementary file 1 — Supporting Information [file ALZ-20-7138-s001.pdf]

Blood-derived microRNAs are related to cognitive domains in the general population

Authors
Konstantinos Melas[1], Valentina Talevi[1], Mohammed Aslam Imtiaz[1], Rika Etteldorf[1], Santiago Estrada[1,2], Dennis M. Krüger[3], Tonatiuh Pena-Centeno[3,4], N. Ahmad Aziz[1,5], Andre Fischer[3,6,7], Monique M.B. Breteler[1,8]

Affiliations
[1] Population Health Sciences, German Centre for Neurodegenerative Diseases (DZNE), Bonn, Germany
[2] AI in Medical Imaging, German Centre for Neurodegenerative Diseases (DZNE), Bonn, Germany
[3] Department for Epigenetics and Systems Medicine in Neurodegenerative Diseases, German Center for Neurodegenerative Diseases, Göttingen, Germany
[4] Bioinformatics Unit, German Centre for Neurodegenerative Diseases, Göttingen, Germany
[5] Department of Neurology, Faculty of Medicine, University of Bonn, Bonn, Germany
[6] Department for Psychiatry and Psychotherapy, University Medical Center Göttingen, Göttingen, Germany
[7] Cluster of Excellence MBExC, University of Göttingen & University Medical Center Goettingen, Göttingen, Germany
[8] Institute for Medical Biometry, Informatics and Epidemiology (IMBIE), Faculty of Medicine, University of Bonn, Bonn, Germany

Supplementary materials

Supplementary Methods S1: MicroRNA and gene expression sequencing and analysis
Supplementary Methods S2: Weighted gene co-expression network analysis
Supplementary Methods S3: MicroRNA target genes and functional enrichment analysis
Supplementary Table S1: Previous studies that identified circulating microRNAs related to cognition, AD or MCI
Supplementary Figure S1: Construction of weighted gene co-expression network
Supplementary Figure S2: Association of microRNA expression with cognitive scores in Model 2 and Model 3
Supplementary Figure S3: Distribution of normalized counts for cognition-related microRNAs.
Supplementary Figure S4: Neuropsychiatric and neurodegenerative disease sensitivity analysis.
Supplementary Figure S5: Stratified analysis for microRNAs inconsistently associated with cognition in our study and in two previous studies
Supplementary Figure S6: Comparison of microRNA expression between the brain and other tissues.
Supplementary Figure S7: MicroRNA expression in cells.
Supplementary Figure S8: Predicted and downregulated microRNA target genes.
Supplementary Figure S9: Enrichment analysis of brain-expressed microRNA target genes.
Supplementary Figure S10: microRNA expression Quantitative Trait Loci (miR-eQTL) analysis
References

Supplementary Methods S1

MicroRNA (miRNA) sequencing and analysis

Sequencing libraries for miRNAs were prepared using NEBNext® small RNA library preparation kit according to manufacturer´s instructions. Quality control of the sequencing was evaluated through FastQC v0.11.9 [1]. Trimmomatic v.0.39 software [2] was used to remove sequencing adapters, low-quality score reads, and reads shorter than 18 base pairs. The alignment and the quantification of miRNAs was performed using the mirdeep2 version 2.0.1.2 software package [3]. Briefly, following the trimming of low-quality score reads, the sequencing reads were aligned to the Human Genome GRCh38.p13 provided by Ensembl, and the expression of known miRNAs was determined by mapping the sequencing reads to predefined human precursor and mature miRNA sequences retrieved from miRBase v22.1 [4].

Gene expression sequencing and analysis

RNA integrity and quantity were assessed through the tapestation RNA assay on a Tapestation4200 instrument (Agilent). 750 ng of total RNA was used to generate NGS libraries for total RNA sequencing using the TruSeq stranded total RNA kit (Illumina) following manufacturer's instructions with Ribo-Zero Globin reduction. We checked library size distribution using D1000 on a Tapestation4200 instrument (Agilent) and quantified the libraries via Qubit HS dsDNA assays (Invitrogen). We clustered the libraries at 250 pM final clustering concentration on the NovaSeq6000 using S2 v1 chemistry (Illumina) in XP mode and sequenced paired-end 2*50 cycles before demultiplexing using bcl2fastq2 v2.20. Quality control of the sequencing was evaluated through FastQC v0.11.9. Following the trimming of low-quality score reads using Trimmomatic v.0.39 software, sequencing reads were aligned to the human reference genome GRCh38.p13 from Ensembl using STAR v2.7.1 [5]. The count matrix was generated with STAR –quantMode GeneCounts using the human gene annotation version GRCh38.101.

Supplementary methods S2

Construction of weighted gene co-expression network

To construct the WGCNA dendrogram, we first calculated pair-wise biweight midcorrelations of miRNA expression. We then chose a soft thresholding power of 7, which satisfied the scale-free topology criterion of $R^2>0.9$ (**Supplementary Figure S1**), and used a signed network to construct an adjacency matrix. Next, we converted the adjacency matrix to a topological matrix, using the topological overlap measure (TOM), and generated a hierarchical clustering tree with 1-TOM as the distance and using the UPGMA algorithm (method=”average”). Using the Hybrid Dynamic Tree Cut method and setting a minimum module size of 5, we thus identified 16 miRNA modules. We set the *deepsplit* parameter to 4, to increase the overall number of clusters and avoid over-clustering.

Identification of module eigengenes and hub miRNAs

WGCNA permits users to reduce the expression of genes in each module to a singule value. This value, called the module (eigen)expression, is defined as the first principal component

derived from Principal Component Analysis (PCA), and is representative of overall miRNA expression within the module. Using this method, we calculated the eigenexpression of each of the miRNA modules identified in our data. Another useful tool offered by WGCNA is the identification of *hub genes* within each module, which are seen as module representatives, or key drivers of module expression and its relation to traits. We defined *hub miRNAs* as miRNAs with *high module membership* and *high significance*, that is, miRNA expression had to be *a)* highly correlated with module eigenexpression (Pearson's $R \geq 0.8$ between miRNA expression and module eigenexpression) and *b*) significantly associated with the same trait as module expression.

Supplementary Methods S3

Identification of miRNA target genes

To identify genes that could be regulated by miRNAs, we examined the association between the expression of miRNAs and their potential target genes. We utilized the *multimir* R package (v.1.12.0) [6] to obtain a list of putative target genes for each miRNA from three online databases: experimentally validated miRNA-target gene interactions were obtained from the MirTarBase [7] database, while predicted miRNA-target gene interactions were obtained from the TargetScan [8] and miRDB [9,10] databases. To ensure the inclusion of all putative miRNA-target genes, the databases were interrogated for all predicted target genes, regardless of database prediction scores. It has been shown that, when predicting miRNA target genes, individual databases might contain many false negative predictions. False negatives can be minimized by using the union of the results of multiple databases [11]. Therefore, we employed the union of the target genes obtained from the three databases. Subsequently, we constructed separate regression models for each miRNA (independent variable) and their putative target genes (dependent variable), adjusting for age, sex, and blood cell counts. Lastly, we filtered for negatively associated target genes (linear regression beta coefficient<0 and P value $\leq .05$), based on the assumption that miRNAs would downregulate the expression of their target genes. We expect that, by applying this functional validation step, we reduced false positive predictions that might have resulted from ignoring prediction scores and employing the union of multiple databases.

Functional enrichment analysis

For the identified target genes we performed a pathway enrichment analysis using the *clusterProfiler* (v. 3.18.1) R Bioconductor package [12]. As we assume that miRNAs in WGCNA modules jointly regulate biological functions, we pooled the target genes of all the miRNAs belonging to the same module. We then conducted an overrepresentation analysis per module, using enrichment terms obtained from the Gene Ontology: Biological Processes (Gene Ontology) [13] database. The universe (background genes) of the overrepresentation analysis was set to the genes measured in our study (n=11,325). As enrichment terms from Gene Ontology tend to be redundant, similar terms were collapsed using the *rrvgo* (v. 1.2.0) R Bioconductor package [14]. Specifically, a semantic similarity matrix of Gene Ontology terms was constructed and similar terms were grouped, selecting the largest term (as defined by the number of genes in each enriched term) to represent each group. A small similarity

threshold of 0.6 was selected to group only highly redundant terms, and the rest of the parameters were left at default settings. The *P* values of the terms within each group were pooled using the Fisher method and multiple testing adjustment according to the Benjamini-Hochberg false discovery rate method was performed for the pooled *P* values.

Supplementary Table S1: Previous studies that identified circulating microRNAs related to cognition, AD or MCI

Study	Study type	Sample size	Biomaterial	Quantification technique	MicroRNAs identified*
Yaqub et al, 2022 [15]	Population-based, cross-sectional	n=1615	Plasma	RNA sequencing	miR-150-5p, miR-19b-3p, miR-20b-5p, miR-122-5p, miR-92a-3p, miR-4732-3p, miR-146a-5p, miR-30a-5p, miR-23b-3p, let-7c-5p, miR-4449, miR-324-3p , miR-6780b-5p, miR-2861, miR-4800-5p , miR-4688, miR-6800-3p , miR-185-5p , miR-3155b, miR-372-3p, miR-6126, miR-8071, miR-4697-5p, miR-7106-5p, miR-187-3p, miR-1322, miR-1275, miR-3141, miR-8078, miR-197-5p, miR-6870-5p, miR-6738-5p, miR-4539, miR-4522, miR-34a-5p, miR-4534<br>(Identified in multi-marker analysis)
Comfort et al, 2022 [16]	Population-based (male participants), cross-sectional and longitudinal	n=530	Plasma	RNA sequencing	Cross-sectional analysis: miR-148a-5p, miR-335-3p<br>Longitudinal analysis: miR-28-3p, miR-152-3p, miR-30e-3p, miR-148b-3p, miR-326, miR-22-3p, miR-221-3p, miR-4732-3p, miR-652-3p, let-7b-5p, miR-342-5p, miR-574-5p, miR-21-5p, miR-320c, miR-340-5p, miR-194-5p, miR-6842-3p, miR-363-3p, miR-584-5p, miR-99a-5p, miR-191-5p, miR-329-3p, miR-215-5p, miR-181a-5p, let-7f-5p, miR-320b, miR-199a-5p, miR-125b-5p, miR-511-5p, miR-6852-5p, miR-431-5p, miR-493-5p, miR-337-3p
Islam et al, 2021 [17]	Population-based, mouse models, case-control	Population-based: n=132;<br>Case-control: n= 71 MCI patients, n=65 HC	Population-based: Whole blood	Population-based: RNA sequencing	miR-181a-5p, miR-146a-5p, miR-148a-3p
Van der Auwera et al, 2023 [18]	Population-based, longitudinal	n = 548	Plasma	qPCR	miR-19a-3p, miR-485-5p
Sadlon et al [19]	Population-based, cross-sectional	n=830	Whole blood	qPCR	let-7a-5p, let-7c-5p, let-7d-5p, miR-144-5p, miR-93-5p, miR-98-5p, miR-363-3p<br>(Identified in linear regression analysis)
Yoon et al, 2022 [20]	Meta-analysis	AD: n=2787 patients, n=2726 HC;<br>MCI: n=302 patients, n=408 HC	Whole blood (2 studies), plasma (6 studies), serum (7 studies), plasma-derived exosomes (2 studies), PBMCs (2 studies), B-lymphocytes (1 study), not specified (19 studies)	qPCR (38 studies), RNA sequencing (1 study), expression assay (1 study)	AD: miR-103a-3p, miR-106a-5p, miR-107, miR-1306-5p, miR-146a-5p, miR-15a-5p, miR-15b-3p, miR-20a-5p, miR-27b-3p, miR-29b-3p, miR-30e-5p, miR-335-5p, miR-361-5p, miR-18b-5p, has-miR-221-3p, miR-31-5p, miR-424-5p, miR-582-5p, miR-9-5p<br>MCI: miR-128-3p, miR-132-3p, miR-323a-3p, miR-134-3p, miR-874-3p<br>(microRNAs differentially expressed in blood with I^2<0.4)

* Underlined microRNAs were measured in the Rhineland Study.
Abbreviations: MCI, Mild Cognitive Impairment; HC, Healthy Control; AD, Alzheimer's Disease

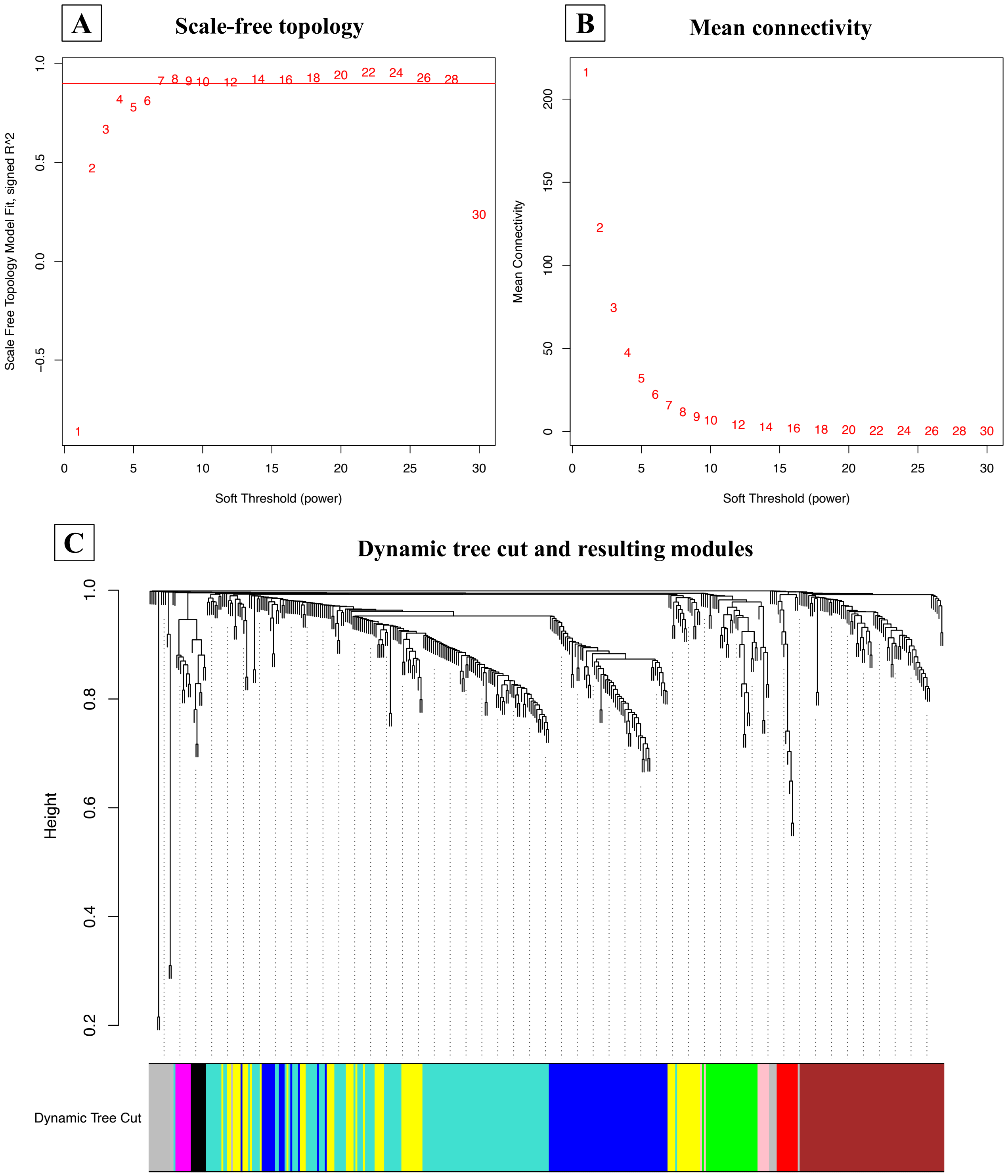

Supplementary Figure S1: Construction of weighted gene co-expression network.
(A) Scale-free topology and **(B)** mean connectivity as a function of soft thresholding power.
(C) Implementation of the dynamic tree cut algorithm and distribution of microRNAs in color-coded clusters (modules). Resulting modules are shown as colors at the lower part of the plot.

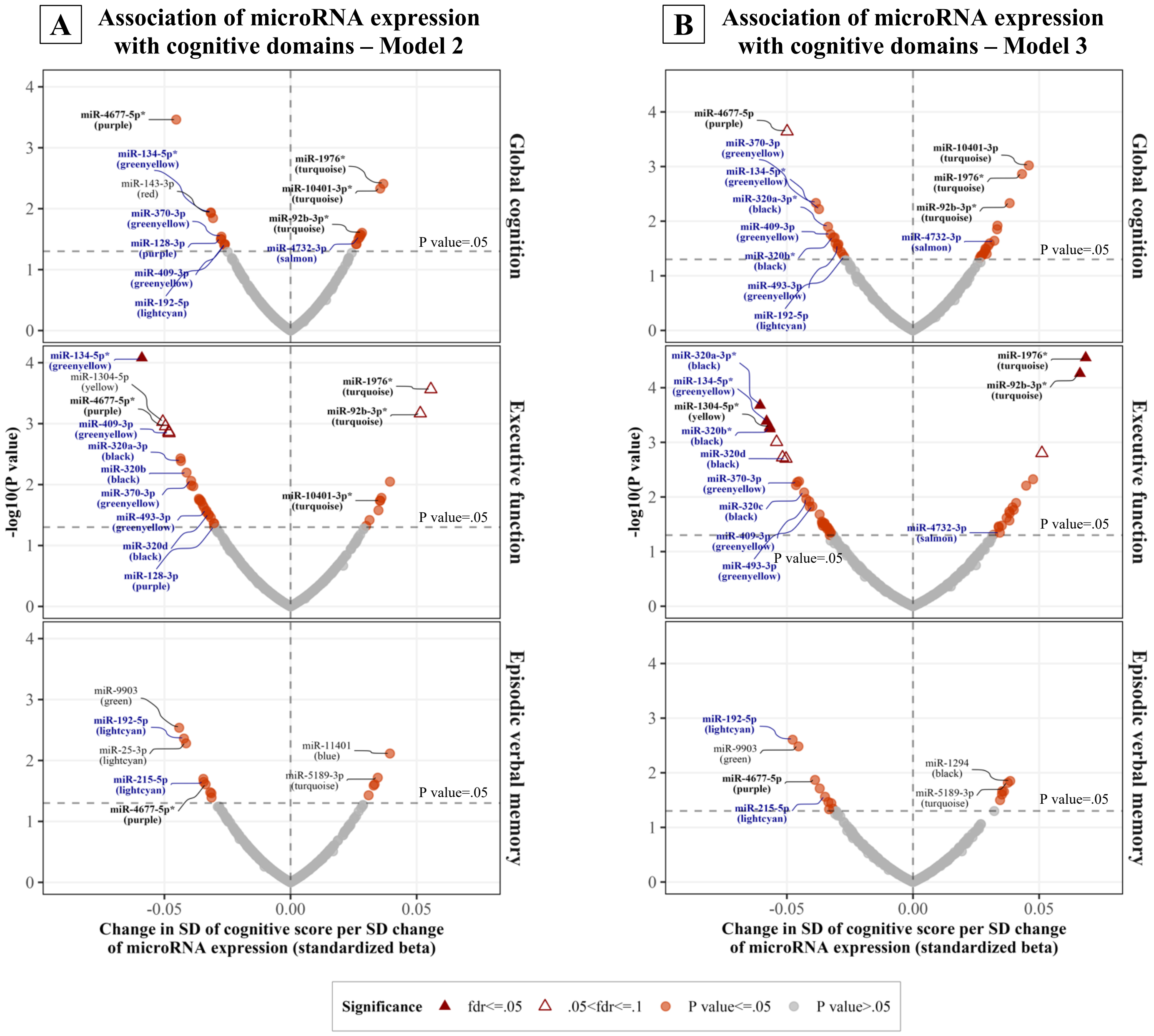

Supplementary Figure S2: Association of microRNA (miRNA) expression with cognitive scores in Model 2 **(A)** and Model 3 **(B).**

Volcano plots of the association of all miRNAs profiled in our study with global cognition, executive function and episodic verbal memory scores. Model 2 has been adjusted for age, sex, educational level and native language. Model 3 has been adjusted for age, sex and blood cell counts. The *P* value shown in the y-axis has not been adjusted for multiple testing. Top miRNAs for each score are annotated and co-expression modules are shown in parentheses. MiRNAs that reached statistical significance for at least one phenotype, after multiple testing correction (fdr ≤ 0.05), are shown with triangles and have been marked with an asterisk (*). Hub miRNAs are annotated with blue color.

Abbreviations: SD, Standard Deviation, fdr, false discovery rate

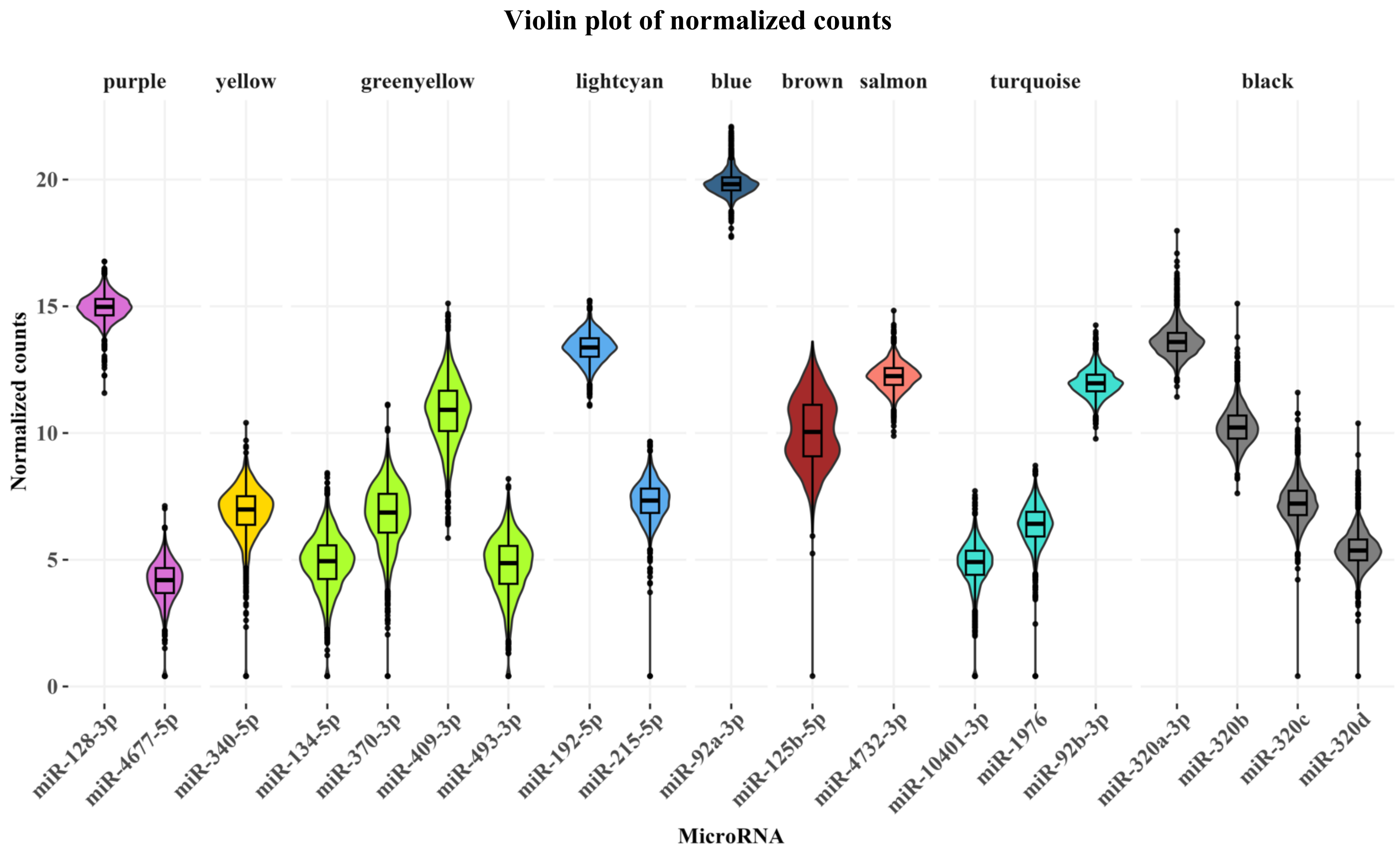

Supplementary Figure S3: Distribution of normalized counts for cognition-related microRNAs (miRNAs).
The boxplots and violin plots show the distribution of DESeq2-normalized counts for the 15 identified cognition-related miRNAs, before adjusting for sequencing batch. MiRNAs have been grouped according to the Weighted Gene Co-expression module they belong to.

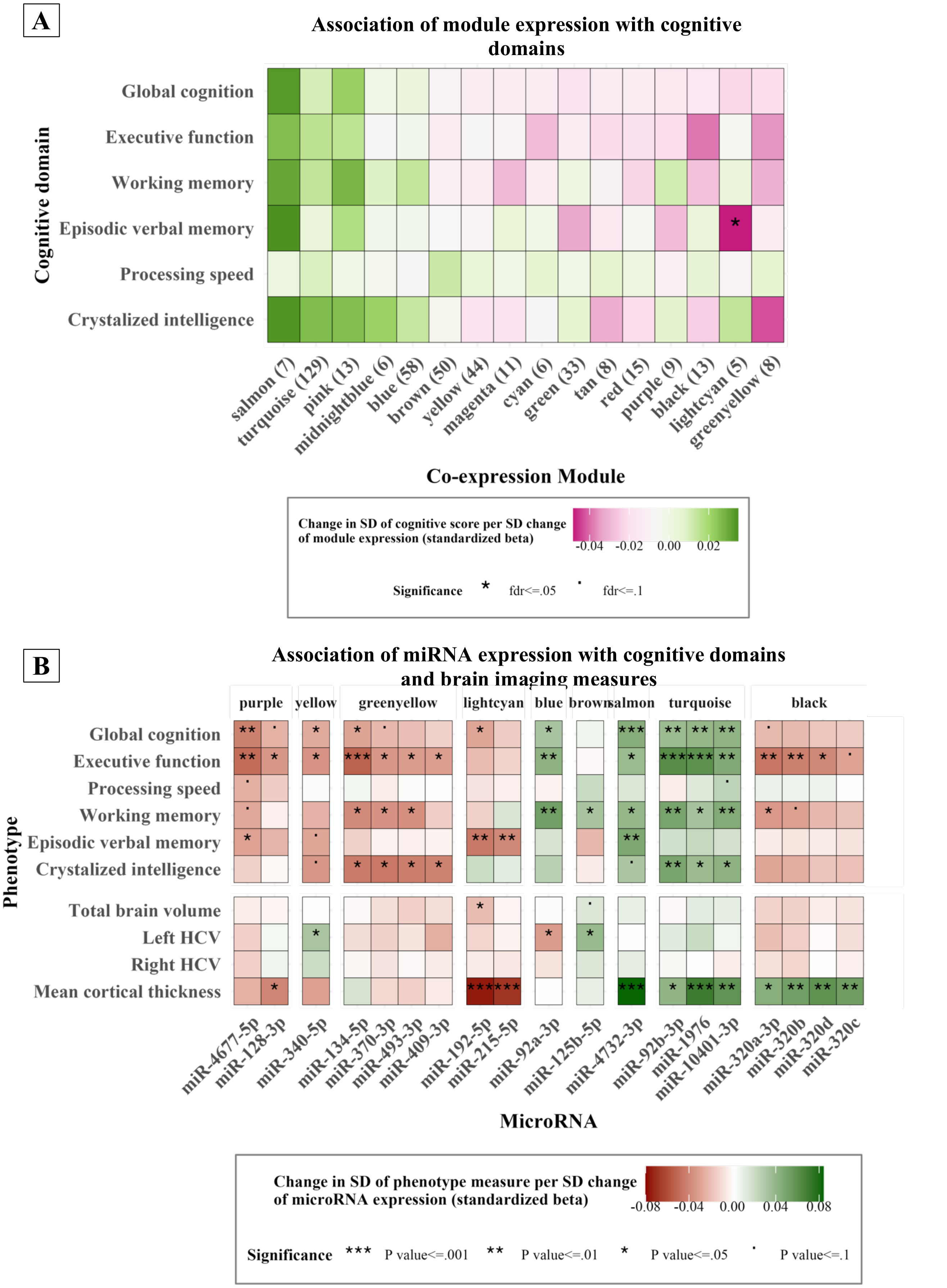

Supplementary Figure S4: Neuropsychiatric and neurodegenerative disease sensitivity analysis. Linear regression coefficients and significance levels for the association of (**A**) co-expression modules and (**B**) individual microRNAs (miRNAs) with cognitive scores, after exclusion of participants who reported a physician diagnosis of dementia, Parkinson's disease, multiple sclerosis, stroke, and schizophrenia.

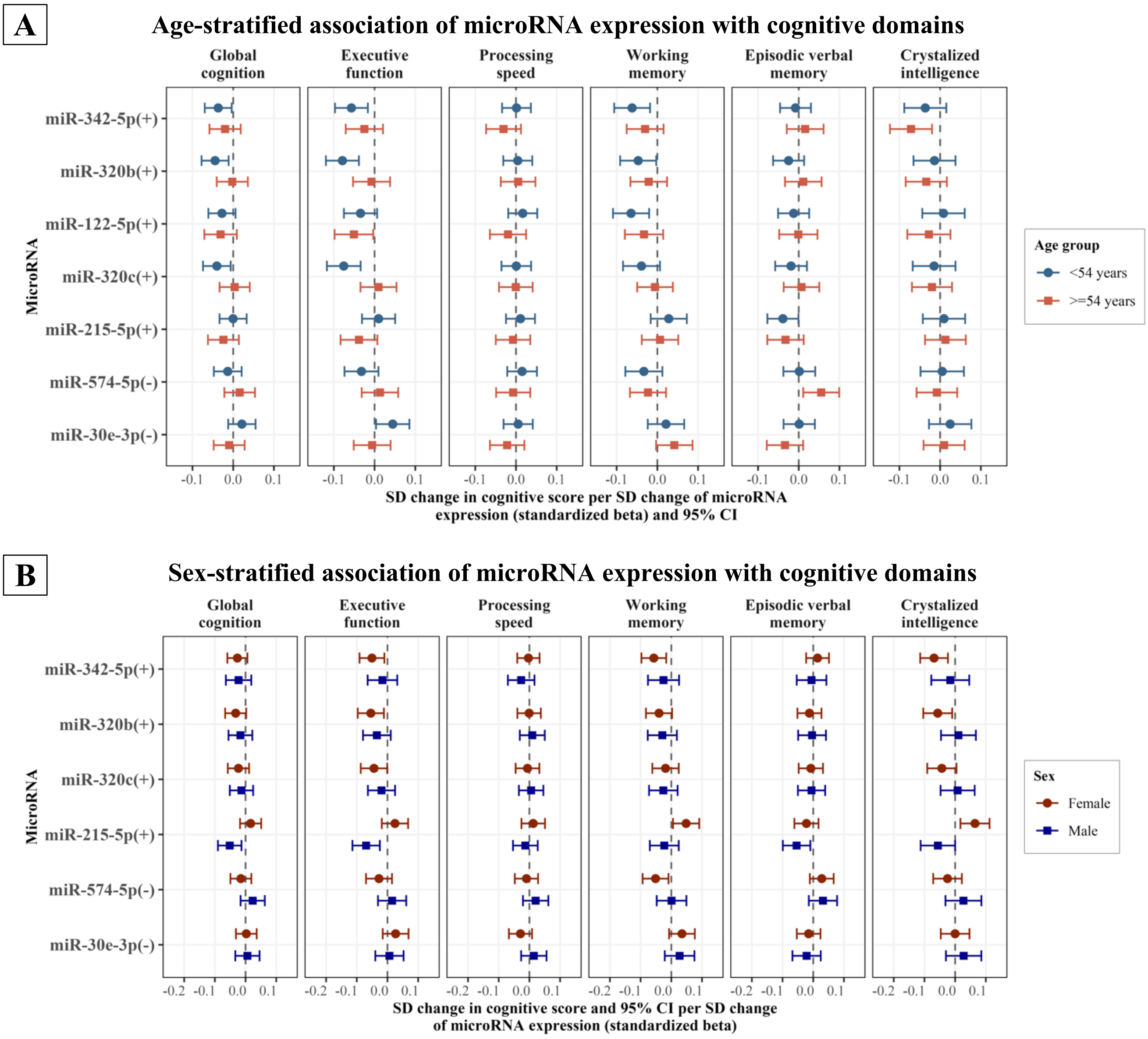

Supplementary Figure S5: Stratified analysis for microRNAs (miRNAs) inconsistently associated with cognition in our study and in previous studies.
Seven miRNAs were associated with cognition in a different direction in our study and in two previous studies [14,15]. The forest plot shows the standardized regression betas and 95% confidence intervals the association of these miRNAs with cognitive scores after stratifying by **(A)** age and (**B**) sex. MiRNAs are marked with “+” when their higher expression was associated with better cognitive function in the previous studies, and with “-” in the opposite case. Linear regression analyses were based on Model 1, which was adjusted for age.
Abbreviations: SD, Standard Deviation; CI, Confidence Interval

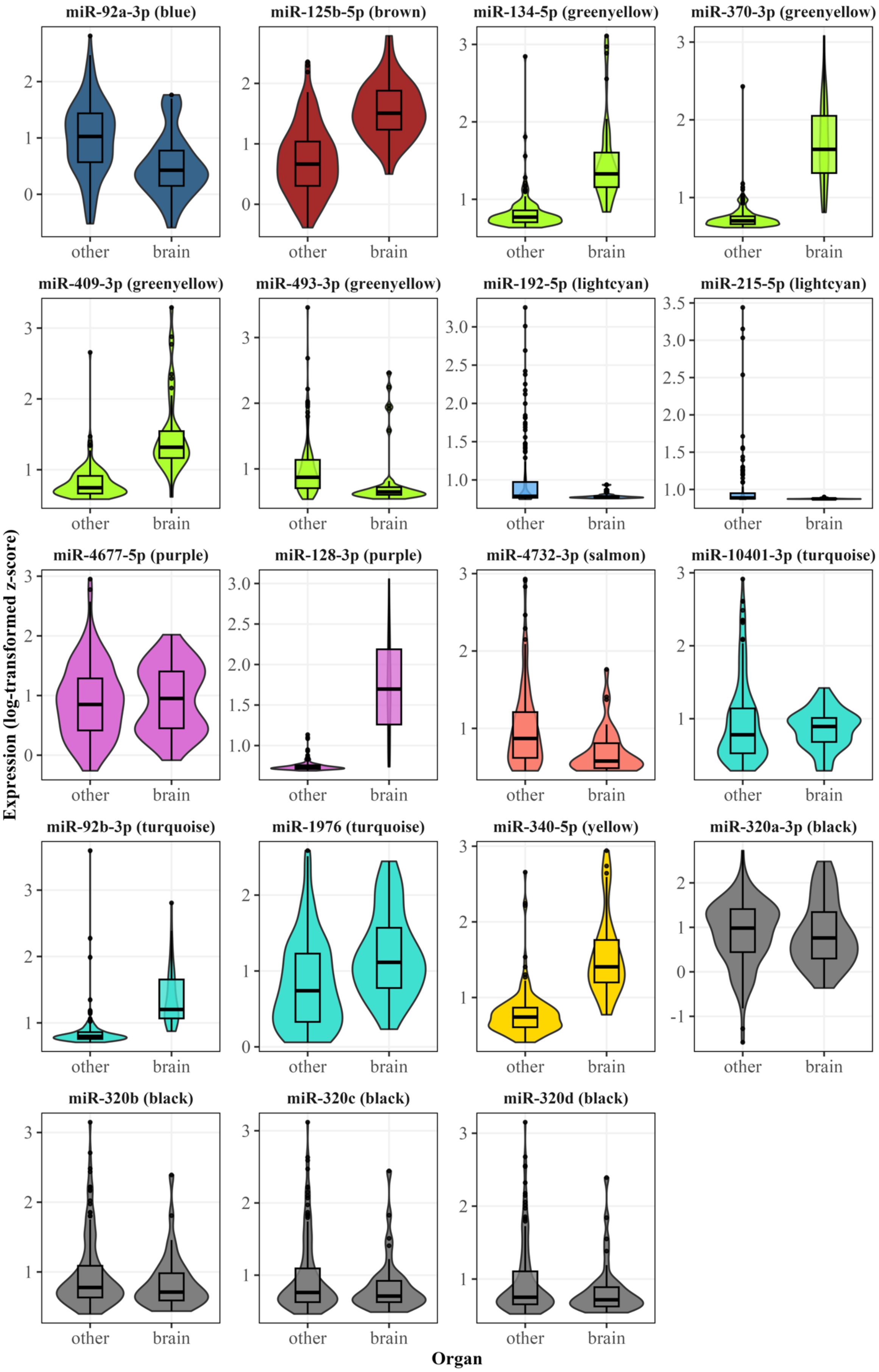

Supplementary Figure S6: Comparison of microRNA (miRNA) expression between the brain and other tissues.

Violin-boxplots of tissue expression, grouped between the brain and other tissues, for miRNAs associated with cognition. Data was obtained from the miRNA Tissue Atlas [21], based on post-mortem samples taken from 6 human donors. The module each miRNA belongs to is shown in parentheses.

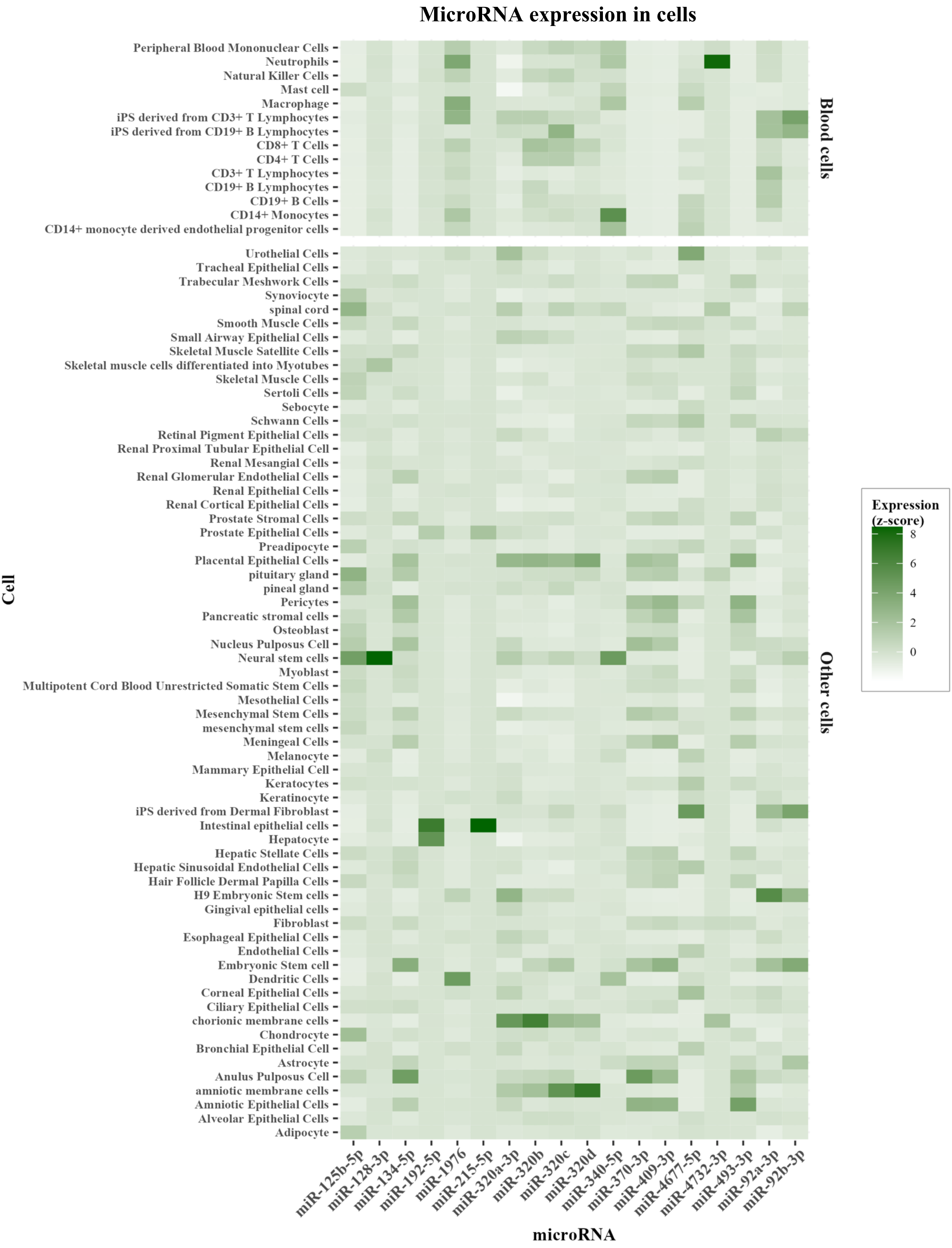

Supplementary Figure S7: MicroRNA (miRNA) expression in cells.
The heatmap shows relative miRNA expression (converted to a z-score for each miRNA) in various cell lines. The heatmap was created using data from a cell expression atlas based on the Functional Annotation of Mammalian Genome (FANTOM5) project [22].

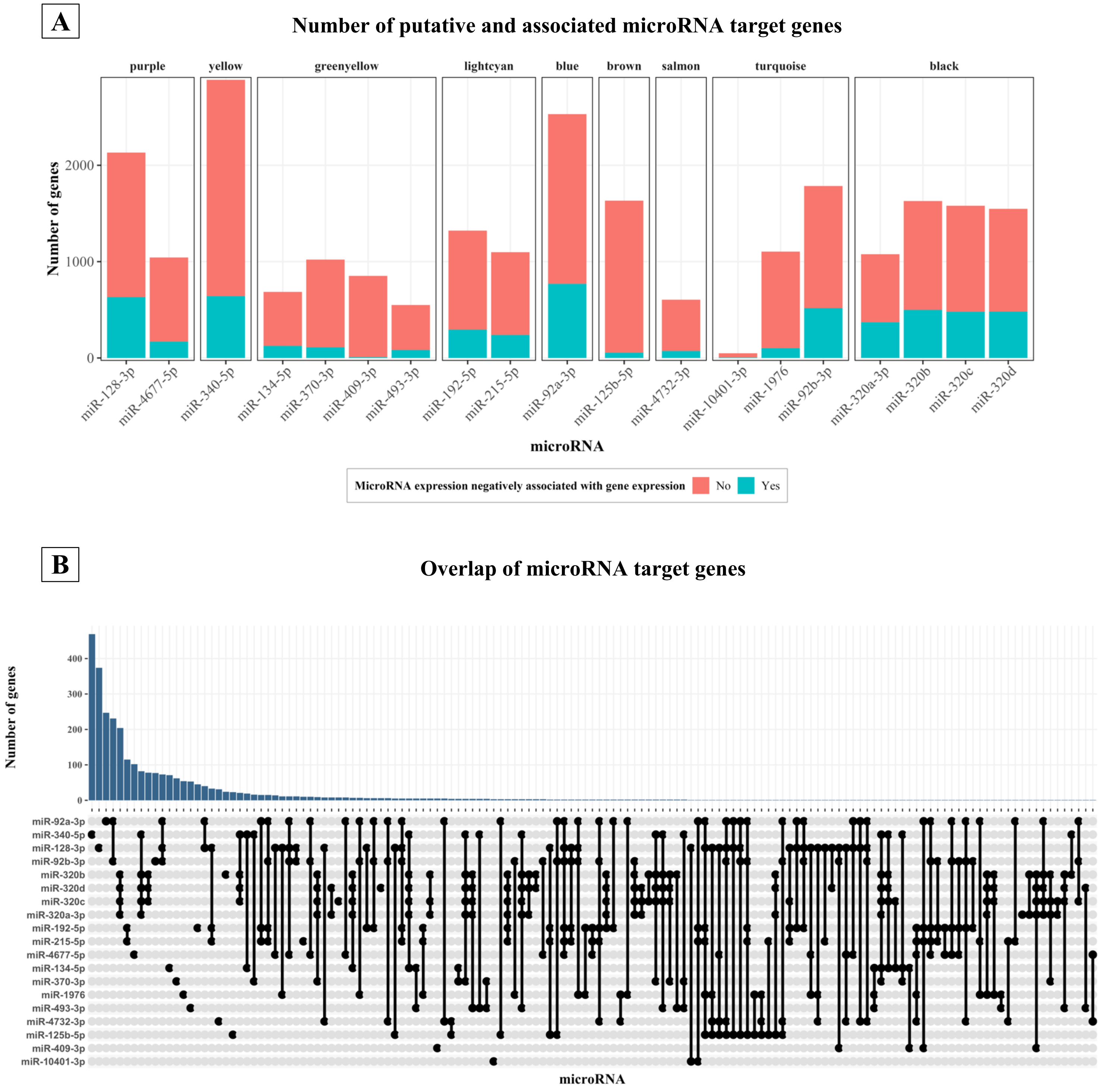

Supplementary Figure S8: Predicted and downregulated microRNA (miRNA) target genes. (**A**) The bar plot shows the number of genes that were predicted to be targeted by miRNAs, and the proportion of these predicted targets the expression of which was negatively associated with the expression of the targeting miRNA. MiRNAs have been grouped by Weighted Gene Co-expression module. (**B**) The upset plot shows the overlap of negatively associated target genes per miRNA. The miRNAs are represented by dots. The lines linking the dots denote that the corresponding miRNAs have common negatively associated target genes. The number of these genes is shown in the bar plot above.

Most enriched pathways for brain-expressed microRNA target genes per co-expression module

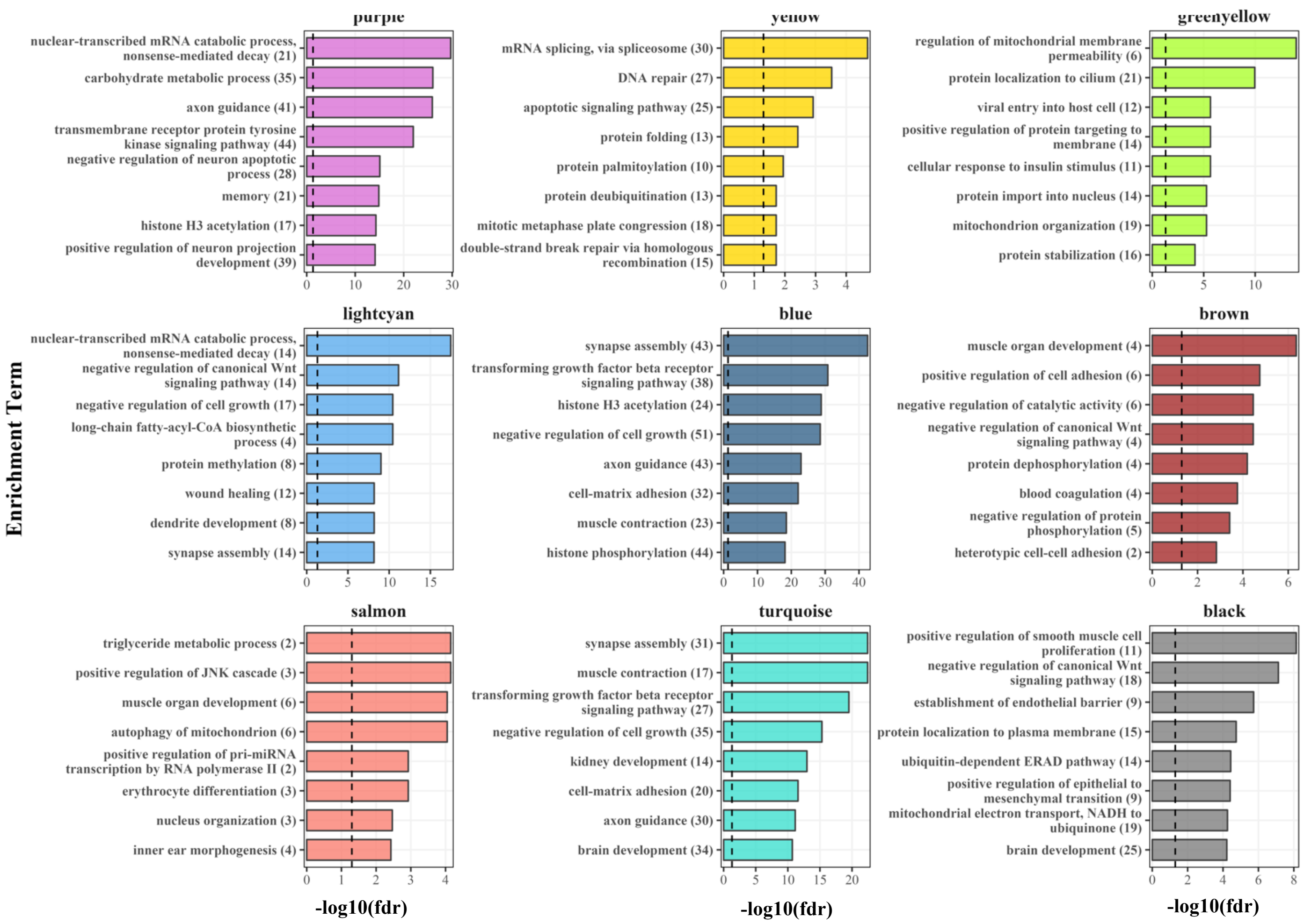

Supplementary Figure S9: Enrichment analysis of brain-expressed microRNA (miRNA) target genes.
Results of *Gene Ontology: Biological Processes* enrichment analysis of negatively associated target genes of the miRNAs in each co-expression module, filtering for genes that are expressed in the brain (normalized Transcript per Million > 10). The top 8 most enriched terms (based on *P* values) are shown. The numbers in parentheses indicate the number of target genes in each enriched term. *P* values have been corrected for multiple testing using the fdr method, with the dash line indicating the fdr = 0.05 threshold. Note that, to allow for better comparison of relative enrichment in each module, the scale of the x-axis (negative logarithm of fdr) varies.
Abbreviations: fdr, false discovery rate

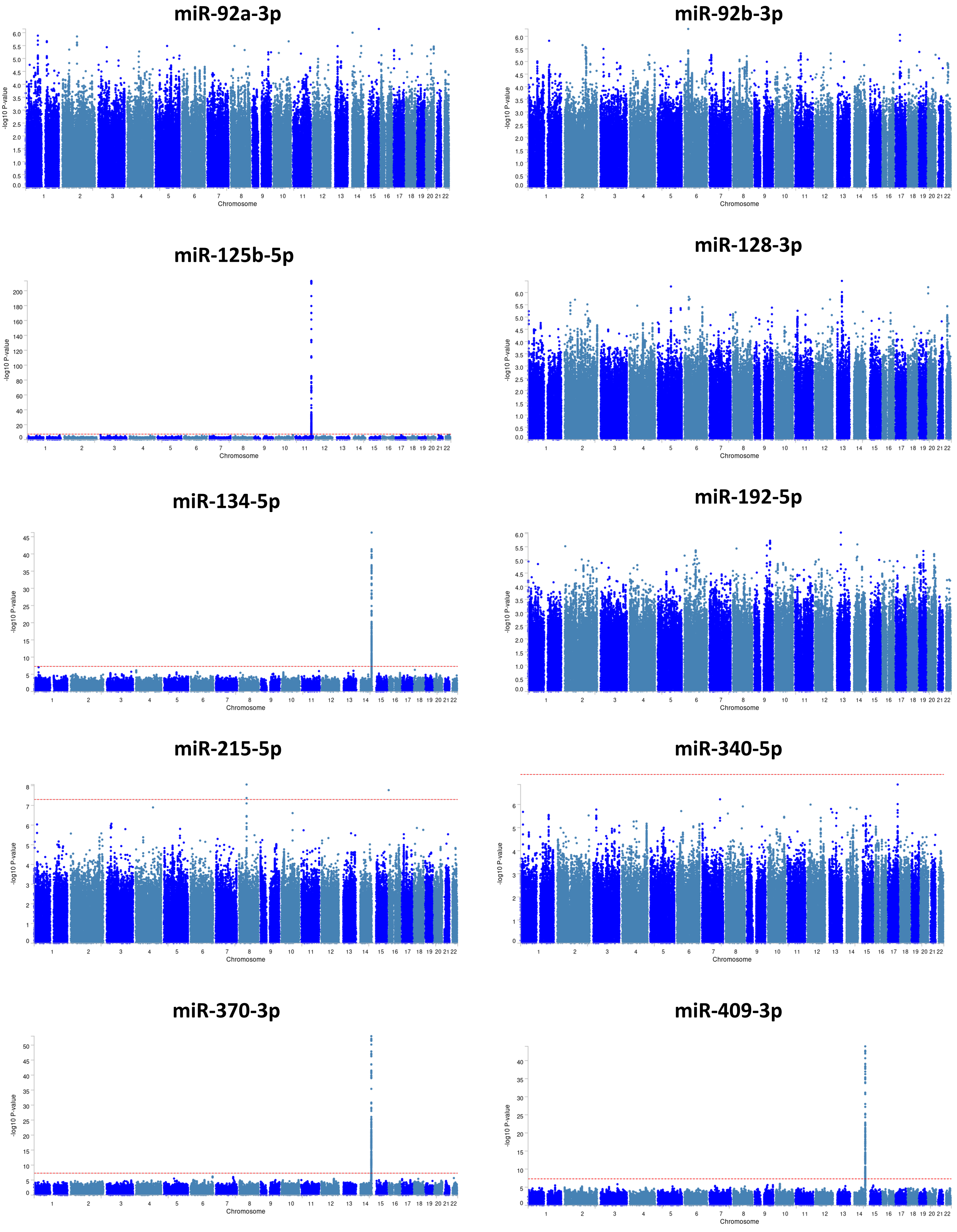

Supplementary Figure S10: MicroRNA (miRNA) expression Quantitative Trait Loci (miR-eQTL) analysis

Manhattan plots of Single Nucleotide Polymorphisms (SNPs) affecting miRNA expression, for each of the 19 cognition-related miRNAs. Each dot represents a signle SNP. The red dashed line represents the P value ≤ 5×10^{-8} significance threshold.

miR-493-3p

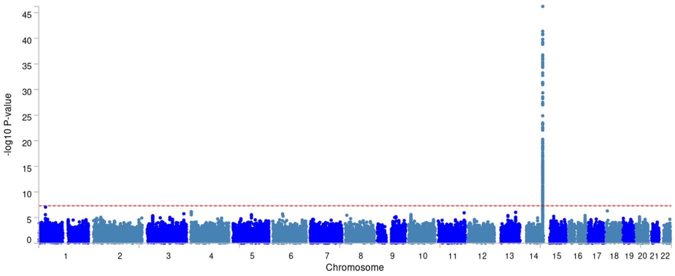

miR-1976

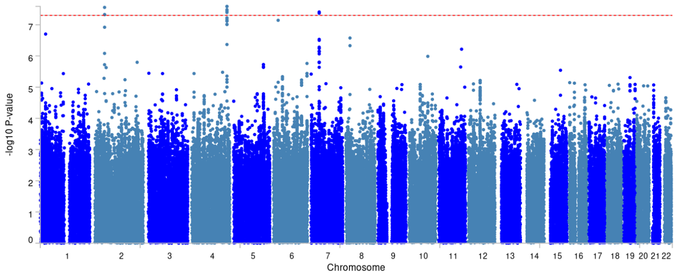

miR-4677

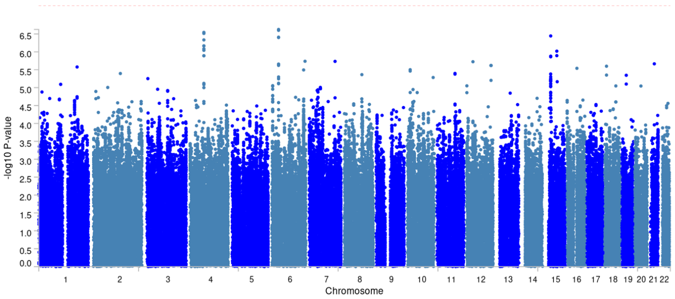

miR-4732-3p

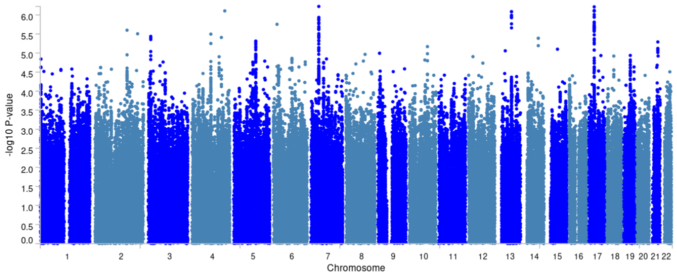

miR-10401-3p

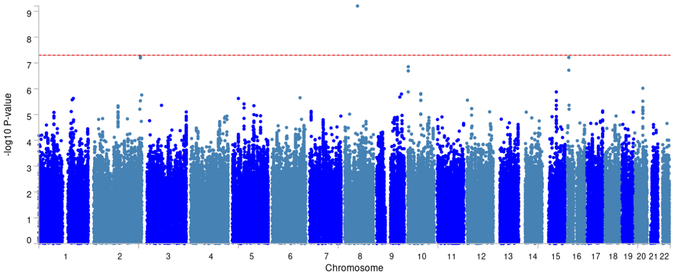

miR-320a-3p

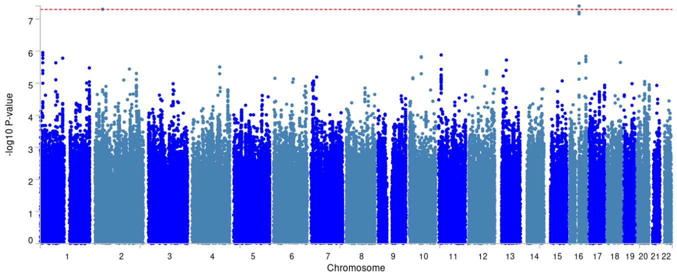

miR-320b

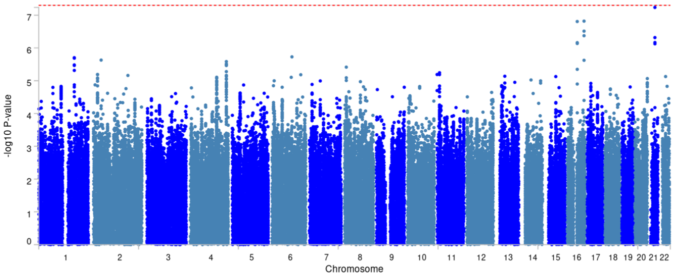

miR-320c

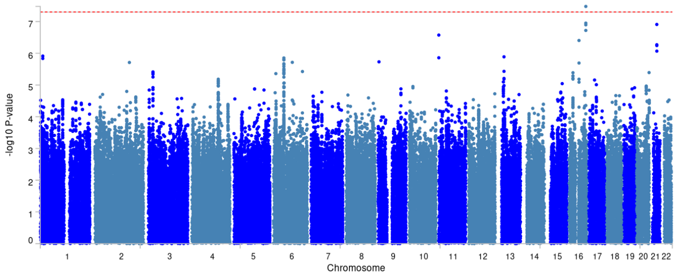

miR-320d

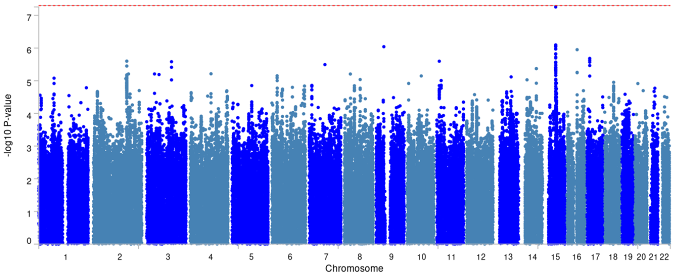

Supplementary Figure S10 (cont.): MicroRNA (miRNA) expression Quantitative Trait Loci (miR-eQTL) analysis
Manhattan plots of Single Nucleotide Polymorphisms (SNPs) affecting miRNA expression, for each of the 19 cognition-related miRNAs. Each dot represents a signle SNP. The red dashed line represents the P value ≤ 5×10^{-8} significance threshold.

References

[1] Andrews S. FastQC: A Quality Control Tool for High Throughput Sequence Data 2010. https://www.bioinformatics.babraham.ac.uk/projects/fastqc/ (accessed November 29, 2022).

[2] Bolger AM, Lohse M, Usadel B. Trimmomatic: A flexible trimmer for Illumina sequence data. Bioinformatics 2014;30:2114–20. https://doi.org/10.1093/bioinformatics/btu170.

[3] Friedländer MR, Mackowiak SD, Li N, Chen W, Rajewsky N. miRDeep2 accurately identifies known and hundreds of novel microRNA genes in seven animal clades. Nucleic Acids Res 2012;40:37–52. https://doi.org/10.1093/nar/gkr688.

[4] Kozomara A, Birgaoanu M, Griffiths-Jones S. miRBase: from microRNA sequences to function. Nucleic Acids Res 2019;47:D155–62. https://doi.org/10.1093/nar/gky1141.

[5] Dobin A, Davis CA, Schlesinger F, Drenkow J, Zaleski C, Jha S, et al. STAR: ultrafast universal RNA-seq aligner. Bioinformatics 2013;29:15–21. https://doi.org/10.1093/bioinformatics/bts635.

[6] Ru Y, Kechris KJ, Tabakoff B, Hoffman P, Radcliffe RA, Bowler R, et al. The multiMiR R package and database: integration of microRNA-target interactions along with their disease and drug associations. Nucleic Acids Res 2014;42:e133. https://doi.org/10.1093/nar/gku631.

[7] Huang H-Y, Lin Y-C-D, Li J, Huang K-Y, Shrestha S, Hong H-C, et al. miRTarBase 2020: updates to the experimentally validated microRNA-target interaction database. Nucleic Acids Res 2020;48:D148–54. https://doi.org/10.1093/nar/gkz896.

[8] McGeary SE, Lin KS, Shi CY, Pham TM, Bisaria N, Kelley GM, et al. The biochemical basis of microRNA targeting efficacy. Science 2019;366. https://doi.org/10.1126/science.aav1741.

[9] Chen Y, Wang X. miRDB: an online database for prediction of functional microRNA targets. Nucleic Acids Res 2020;48:D127–31. https://doi.org/10.1093/nar/gkz757.

[10] Liu W, Wang X. Prediction of functional microRNA targets by integrative modeling of microRNA binding and target expression data. Genome Biol 2019;20:18. https://doi.org/10.1186/s13059-019-1629-z.

[11] Oliveira AC, Bovolenta LA, Nachtigall PG, Herkenhoff ME, Lemke N, Pinhal D. Combining Results from Distinct MicroRNA Target Prediction Tools Enhances the Performance of Analyses. Front Genet 2017;8:59. https://doi.org/10.3389/fgene.2017.00059.

[12] Wu T, Hu E, Xu S, Chen M, Guo P, Dai Z, et al. clusterProfiler 4.0: A universal enrichment tool for interpreting omics data. Innovation (Camb) 2021;2:100141. https://doi.org/10.1016/j.xinn.2021.100141.

[13] The Gene Ontology Consortium. The Gene Ontology resource: enriching a GOld mine. Nucleic Acids Res 2021;49:D325–34. https://doi.org/10.1093/nar/gkaa1113.

[14] Sayols S. rrvgo: a Bioconductor package for interpreting lists of Gene Ontology terms. MicroPubl Biol 2023;2023. https://doi.org/10.17912/micropub.biology.000811.

[15] Yaqub A, Mens MMJ, Klap JM, Weverling GJ, Klatser P, Brakenhoff JPJ, et al. Genome-wide profiling of circulatory microRNAs associated with cognition and dementia. Alzheimers Dement 2022. https://doi.org/10.1002/alz.12752.

[16] Comfort N, Wu H, De Hoff P, Vuppala A, Vokonas PS, Spiro A, et al. Extracellular microRNA and cognitive function in a prospective cohort of older men: The Veterans Affairs Normative Aging Study. Aging (Albany NY) 2022;14:6859–86. https://doi.org/10.18632/aging.204268.

[17] Islam MR, Kaurani L, Berulava T, Heilbronner U, Budde M, Centeno TP, et al. A microRNA signature that correlates with cognition and is a target against cognitive decline. EMBO Mol Med 2021;13:e13659. https://doi.org/10.15252/emmm.202013659.

[18] Van der Auwera S, Garvert L, Ameling S, Völzke H, Nauck M, Völker U, et al. The interplay between micro RNAs and genetic liability to Alzheimer’s Disease on memory trajectories in the general population. Psychiatry Res 2023;323:115141. https://doi.org/10.1016/j.psychres.2023.115141.

[19] Sadlon A, Takousis P, Evangelou E, Prokopenko I, Alexopoulos P, Udeh-Momoh CM, et al. Association of Blood MicroRNA Expression and Polymorphisms with Cognitive and Biomarker Changes in Older Adults. J Prev Alzheimers Dis 2024;11:230–40. https://doi.org/10.14283/jpad.2023.99.

[20] Yoon S, Kim SE, Ko Y, Jeong GH, Lee KH, Lee J, et al. Differential expression of MicroRNAs in Alzheimer’s disease: a systematic review and meta-analysis. Mol Psychiatry 2022;27:2405–13. https://doi.org/10.1038/s41380-022-01476-z.

[21] Keller A, Gröger L, Tschernig T, Solomon J, Laham O, Schaum N, et al. miRNATissueAtlas2: an update to the human miRNA tissue atlas. Nucleic Acids Res 2022;50:D211–21. https://doi.org/10.1093/nar/gkab808.

[22] de Rie D, Abugessaisa I, Alam T, Arner E, Arner P, Ashoor H, et al. An integrated expression atlas of miRNAs and their promoters in human and mouse. Nat Biotechnol 2017;35:872–8. https://doi.org/10.1038/nbt.3947.
